# Multi-omics and spatial transcriptomics decode the ZDHHC9-driven hypoxia-immunosuppressive axis in hepatocellular carcinoma

**DOI:** 10.3389/fonc.2026.1869712

**Published:** 2026-06-17

**Authors:** Haiyan Lu, Lingzhen Kong, Shidong Hu, Wenyuan Xie, Yi Wang, Jianhua Wang, Fengsheng Dai

**Affiliations:** 1 Department of Oncology, Renji Hospital, School of Medicine, Chongqing University, The Fifth People’s Hospital of Chongqing, Chongqing, China; 2 Department of Oncology, Nanxishan Hospital of The Guangxi Zhuang Autonomous Region (Second People’s Hospital of Guangxi Zhuang Autonomous Region), Guilin, Guangxi, China; 3 Department of Spine Surgery, Zhongda Hospital Southeast University, Nanjing, China; 4 Department of Hepatobiliary Pancreatic Tumor Center, Chongqing University Cancer Hospital, Chongqing, China; 5 Department of Oncology, The First Affiliated Hospital of Dali University, Dali, China

**Keywords:** hepatocellular carcinoma, immune infiltration, palmitoylation, prognosis marker, ZDHHC9

## Abstract

**Background:**

Hepatocellular carcinoma (HCC) is a major global health challenge with limited treatment options, highlighting the urgent need for new biomarkers and therapeutic targets. Protein palmitoylation, mediated by ZDHHC enzymes, plays a role in cancer, yet its comprehensive function in HCC development is not fully understood.

**Methods:**

We conducted a systematic analysis of the ZDHHC family in HCC. Using data from TCGA and GEO databases, we assessed their expression, prognostic value, association with immune infiltration, and drug sensitivity. Diagnostic biomarkers were identified using ten machine learning algorithms. We then developed a consistent machine learning framework to build a robust multi-gene prognostic signature. The tumor microenvironment was further characterized through integrated single-cell and spatial transcriptomic analyses. The oncogenic role of our primary candidate, ZDHHC9, was functionally tested using siRNA knockdown, *in vitro* assays, and an *in vivo* xenograft model.

**Results:**

Our multi-omics analysis pinpointed ZDHHC9 as both a key prognostic factor and the top diagnostic biomarker. We successfully constructed and validated a powerful multi-gene prognostic signature across independent patient cohorts. High ZDHHC9 expression was associated with an immunosuppressive tumor microenvironment and increased therapy resistance. Single-cell and spatial transcriptomics revealed that ZDHHC9 is specifically upregulated in malignant epithelial cells, especially within hypoxic subpopulations. Pan-cancer analysis further confirmed that ZDHHC9 is frequently dysregulated and prognostically relevant in other cancer types. Functionally, depleting ZDHHC9 significantly inhibited HCC cell proliferation, migration, and invasion *in vitro*, and suppressed tumor growth *in vivo*.

**Conclusion:**

This study provides a comprehensive profile of the ZDHHC family in HCC, establishes a robust prognostic model, and nominates ZDHHC9 as a novel diagnostic and prognostic biomarker as well as a promising therapeutic target. The oncogenic function of ZDHHC9 appears to be linked to its role in promoting a hypoxic phenotype and fostering an immunosuppressive microenvironment.

## 1 Introduction

Hepatocellular carcinoma (HCC) continues to be a significant worldwide health challenge, maintaining its status as one of the most frequently diagnosed and fatal cancers globally (1). The epidemiology of HCC displays marked geographical variation, with chronic hepatitis B infection constituting the principal etiological factor in endemic areas. In contrast, metabolic dysfunction-associated steatotic liver disease is contributing to an increasing fraction of cases in Western populations (2, 3). Clinically, managing this malignancy presents considerable difficulties, stemming largely from its common detection at advanced stages, the restricted range of effective systemic therapies available, and the profoundly immunosuppressive tumor microenvironment (TME) that drives therapy resistance and poor prognosis (4–6). These persistent clinical limitations highlights the urgent need for enhanced biomarkers enabling earlier detection and for the development of novel, targeted therapeutic approaches capable of reprogramming the hostile TME to alter the course of this aggressive disease.

Protein post-translational modifications (PTMs) such as phosphorylation, ubiquitination, acetylation, and methylation have become central to understanding cell signaling and disease pathogenesis over the past decades (7–9). Critically, the successful clinical application of kinase inhibitors and proteasome inhibitors in cancer therapy has robustly demonstrated the immense value of targeting PTM-regulating enzymes (10, 11). However, beyond these classical PTMs, protein palmitoylation-a lipid modification catalyzed by DHHC-type containing (ZDHHC) family palmitoyltransferases (PATs) that covalently attaches a 16-carbon palmitate to cysteine residues of substrate proteins-remains a relatively underexplored frontier.

Similar to phosphorylation, palmitoylation is dynamically reversible, finely tuned by PATs and depalmitoylases, and acts as a fast, reversible signaling “switch.” More importantly, palmitoylation anchors target proteins firmly to cell membranes, thereby regulating their subcellular localization, stability, protein-protein interactions, and downstream signaling – a unique regulatory mode not offered by other PTMs (12). In mammals, this reaction is catalyzed by enzymes belonging to the zinc finger ZDHHC palmitoyltransferase family (13, 14). The human genome encodes 23 members of this family, each containing a cysteine-rich domain that harbors a distinctive DHHC signature motif. This domain also possesses the capacity to bind two zinc ions (Zn^2+^), leading to its classification as ZDHHC palmitoyltransferase. Current evidence indicates that palmitoylation of the membrane protein claudin-3 (CLDN3) enhances its localization to the cell membrane and promotes its stability in ovarian cancer cells. Disruption of ZDHHC12 expression reduces CLDN3 palmitoylation, thereby inhibiting its membrane trafficking and curbing tumor progression (15). Notably, palmitoylation directly regulates the stability, trafficking, and function of key immune checkpoint proteins, positioning the ZDHHC family as potential modulators of the tumor immune microenvironment (12). Within the context of HCC, studies have shown that ZDHHC20-mediated palmitoylation of fatty acid synthase (FASN) facilitates hepatocarcinogenesis (16). Furthermore, palmitoylation catalyzed by ZDHHC3 has been implicated in promoting cholesterol synthesis and enabling immune escape in HCC (17). In addition, some studies have preliminarily suggested the correlation of certain ZDHHC genes with HCC patient prognosis through bioinformatics analysis, while other basic experiments have focused on single genes, preliminarily revealing their pro- or anti-tumor functions. However, most of these studies are limited to the investigation of a single or a few ZDHHC genes, lacking a systematic, multi-omics panoramic analysis of the entire ZDHHC family in HCC. In particular, there are very few research reports on ZDHHC9 in HCC; its expression pattern, clinical significance, role in immune microenvironment regulation, and potential as a therapeutic target remain largely unknown, constituting a significant knowledge gap.

In summary, although ZDHHC-mediated protein palmitoylation plays an increasingly recognized role in cancer, its panoramic functional landscape in HCC, the specific role of key effectors like ZDHHC9 in remodeling the tumor microenvironment, and their cell-type specificity remain largely unexplored. Therefore, this study aims to directly address the following central scientific question: How does the ZDHHC family, particularly ZDHHC9, drive tumor progression by remodeling the immune and hypoxic microenvironment of HCC through regulating palmitoylation? To answer this, we integrated multi-dimensional data including bulk RNA-seq, single-cell transcriptomics, and spatial transcriptomics to systematically delineate the expression profile, clinical relevance, functional mechanisms, and spatiotemporal associations with microenvironmental features of ZDHHC9 in HCC, hoping to provide novel targets and theoretical foundations for precise HCC therapy.

## 2 Materials and methods

### 2.1 Acquisition and preprocessing of data

RNA sequencing data for liver hepatocellular carcinoma (LIHC) were obtained from The Cancer Genome Atlas (TCGA) database. Corresponding patient clinical information and immune infiltration data accessible within the TCGA repository were also retrieved. The expression data, initially provided in fragments per kilobase of transcript per million mapped reads (FPKM) format, were subsequently converted to transcripts per million (TPM) and subjected to a log2 transformation for downstream analytical procedures.

### 2.2 Machine learning-based identification of feature genes

To systematically and robustly identify potential diagnostic biomarkers for HCC, we employed ten different machine learning algorithms to analyze the training set data. These algorithms encompassed various modeling philosophies, including: penalized regression models (Least Absolute Shrinkage and Selection Operator (LASSO) regression, Elastic-Net), tree ensemble models (Random Forest (RF), Gradient Boosting Machine (GBM), eXtreme Gradient Boosting (XGBoost)), Support Vector Machine (SVM) and its variants, Naïve Bayes (NB), K-Nearest Neighbors (KNN), and Artificial Neural Network (ANN) (18–22).

All models employed 5-fold repeated cross-validation for parameter optimization and generalization performance assessment. Model performance was evaluated by calculating the area under the receiver operating characteristic curve (AUC) with the pROC package, and the results were visualized for comparative analysis. The contribution of each gene to model predictions was quantified using the DALEX package, with root mean square error serving as the loss function for feature importance ranking (23).

### 2.3 Single-cell RNA sequencing data processing and analysis

To systematically characterize the tumor microenvironment (TME) of hepatocellular carcinoma (HCC) and ensure the robustness of our findings, we analyzed two independent HCC single-cell RNA sequencing (scRNA-seq) datasets (GSE299340 and GSE290298) obtained from the Gene Expression Omnibus (GEO) database (24, 25). Raw data were processed using the Seurat package (v4.0+) in R. Stringent quality control was applied: cells were retained if they expressed between 200 and an upper limit of genes (to exclude potential doublets) and were filtered out if they exhibited an excessively high percentage of mitochondrial genes.

After performing initial quality control and log-normalization separately for each dataset, the Harmony algorithm was employed to correct for technical batch effects arising from different sequencing batches and sample origins. Using these sources of variation as covariates, cells were aligned in the low-dimensional principal component analysis (PCA) space to generate corrected embeddings for subsequent analysis.

Based on the Harmony-corrected principal components, we performed nonlinear dimensionality reduction and visualization using the UMAP algorithm. Unsupervised clustering was conducted via the Louvain algorithm based on a K-nearest neighbor graph. Differentially expressed genes for each cluster were identified using the Find All Markers function (Wilcoxon rank-sum test). Combined with established cell marker genes, the clusters were annotated into six major types: Hepatocytes/Malignant Epithelial cells (markers: ALB, GPC3), T/NK cells (CD3D, NKG7), Myeloid cells (CD68, C1QA), B/Plasma cells (CD79A, IGHG1), Fibroblasts (COL1A1, PDGFRA), and Endothelial cells (VWF, PECAM1).

To investigate the heterogeneity of tumor epithelial cells, the “Hepatocytes/Malignant Epithelial” cell population was extracted for re-analysis, including highly variable gene selection, dimensionality reduction, and fine subclustering. Based on their specific gene expression profiles, they were subdivided into functional subtypes: Metabolic, Proliferating, Hypoxic/Stressed, EMT/Invasive, and Stem-like/Resistant. To confirm their malignant nature at single-cell resolution, inferred copy number variation (infer CNV) analysis was performed: using hepatocytes from normal tissues as a diploid reference, genome-wide copy number alterations were inferred from gene expression profiles, and a “Genomic Instability Score” was calculated for each cell.

Based on the integrated data, the proportional distribution of each cell type and epithelial subpopulation in normal versus tumor tissues was calculated, and differences between groups were compared using the Wilcoxon rank-sum test. To evaluate palmitoylation modification activity, we utilized the complete set of ZDHHC family genes. The Add Module Score function in Seurat (with randomly selected background genes as a control) was used to calculate a “Palmitoylation Composite Score” for each cell, and this score was compared across different cell populations.

### 2.4 Cell lines and culture conditions

The human HCC cell lines Hep3B and Huh7 were obtained from the American Type Culture Collection (ATCC, Manassas, VA, USA). Cells were cultured in RPMI-1640 medium (Gibco-BRL, Germany) supplemented with 10% fetal bovine serum (FBS; Biological Industries, USA). All cell cultures were maintained at 37 °C in a humidified incubator with 5% CO_2_.

### 2.5 Small interfering RNA transfection

siRNA sequences targeting ZDHHC9 (si-ZDHHC9#1: 5’-AGAAGAUGUCAGUCACCUCUGAUA-3’; si-ZDHHC9#2: 5’-GUAGAGAUAAUAAUUGACAUUUCTC-3’) were synthesized by GenePharma (Shanghai, China). Transfection procedures were carried out as described previously (26). Cells were harvested for subsequent experiments 48 to 72 hours post-transfection.

### 2.6 Western blot

Protein expression was assessed by western blotting as described previously (27). The primary antibodies used in this study were ZDHHC9 (ER63246; HUABIO) and β-actin (sc-47778; Santa Cruz). Corresponding secondary antibodies, Goat Anti-Mouse IgG (BL001A; Biosharp) and Goat Anti-Rabbit IgG (BL003A; Biosharp), were applied for detection. Protein bands were visualized using an enhanced chemiluminescence (ECL) reagent. All assays were performed in three independent replicates.

### 2.7 Cell proliferation, colony formation, migration, and invasion assays

Cell proliferation, colony formation, and transwell assays were performed according to established protocols (26, 27). For the CCK-8 assay, Hep3B and Huh7 cells were seeded into 96-well plates at a density of 1,000 cells per well in 100 μL of RPMI-1640 medium containing 10% FBS. Cell viability was measured at 24, 48, 72, and 96 hours using a CCK-8 kit (Promega). In the colony formation assay, a limited number of cells were plated into six-well plates. After approximately 14 days of incubation to allow colony development, cells were fixed with 4% paraformaldehyde and stained with 0.1% crystal violet.

Cell migration and invasion were evaluated using transwell chambers (Corning Incorporated) with an 8 μm pore size. To assess invasion, the transwell membrane was pre-coated with Matrigel (BD Biosciences). Following fixation and staining, cells that had migrated or invaded to the lower side of the membrane were imaged by phase-contrast microscopy and counted. Each experiment was conducted in triplicate.

### 2.8 *In vivo* xenograft model

All procedures involving animals received approval from the Animal Ethics Committee of Chongqing University Cancer Hospital. Female BALB/c nude mice (6 weeks old; n=3 per group) were housed under specific pathogen-free (SPF) conditions. For tumor establishment, a single-cell suspension comprising 4×10^6^ Hep3B cells in 200 μL of serum-free DMEM was delivered via subcutaneous injection into the right flank of each mouse. Treatment was initiated with intratumoral injections of either control siRNA (si-NC) or ZDHHC9-targeting siRNA (10 nmol in 0.1 mL saline per tumor), administered every 48 hours for a total of six doses. Tumor size was measured starting on day 7 post-inoculation, with volume calculated using the formula V = (length × width²)/2. On day 19, mice were euthanized by gradual displacement of chamber air with compressed CO_2_, followed by cervical dislocation to confirm death. Tumors were excised and weighed.

### 2.9 Analysis of spatial transcriptomic data

Spatial transcriptomic data derived from HCC tissue sections were obtained from the published work of Liu et al (28). Analysis of tissue sections was conducted using the Sparkle database (https://www.grswsci.top/analyze/), a resource that utilizes established analytical frameworks (29, 30) and integrates 10x Visium sequencing to produce detailed spatial transcriptomic maps of HCC.

The raw gene expression count matrix was downloaded from GEO and subsequently processed using the Seurat package (v4.0+). First, the gene expression data for each spatial spot was normalized using the NormalizeData function to obtain standardized expression values. Then, based on the cell composition results provided by the authors of the dataset, which were calculated via deconvolution analysis, we annotated each spatial spot: spots with a malignant cell proportion greater than 0 were defined as malignant regions (Malignant, Mal), while those with a proportion equal to 0 were defined as non-malignant regions (Non-malignant, nMal). Following region definition, differential gene expression between Mal and nMal regions was statistically assessed using the wilcox.test function in R. Furthermore, Spearman correlation analysis was applied to examine the correlations in cellular composition across all spatial spots, as well as the relationships between specific gene expression levels and cellular abundances.

### 2.10 Statistical analysis

All datasets from TCGA were analyzed using R software (version 4.2.1). Differences in gene expression between HCC tumors and adjacent normal tissues were assessed using the Wilcoxon rank-sum test (for unpaired groups) and the Wilcoxon signed-rank test (for paired samples). Kaplan-Meier survival curves were generated with the survival package. For all analyses involving patient stratification by ZDHHC9 expression, patients were dichotomized into high and low expression groups using the median expression value of ZDHHC9 in the specific cohort as the cut-off point. A two-tailed test was employed for all analyses, with a p-value < 0.05 considered statistically significant.

## 3 Results

### 3.1 A consistent machine learning framework identifies a robust ZDHHC prognostic signature

**Figure 1:**
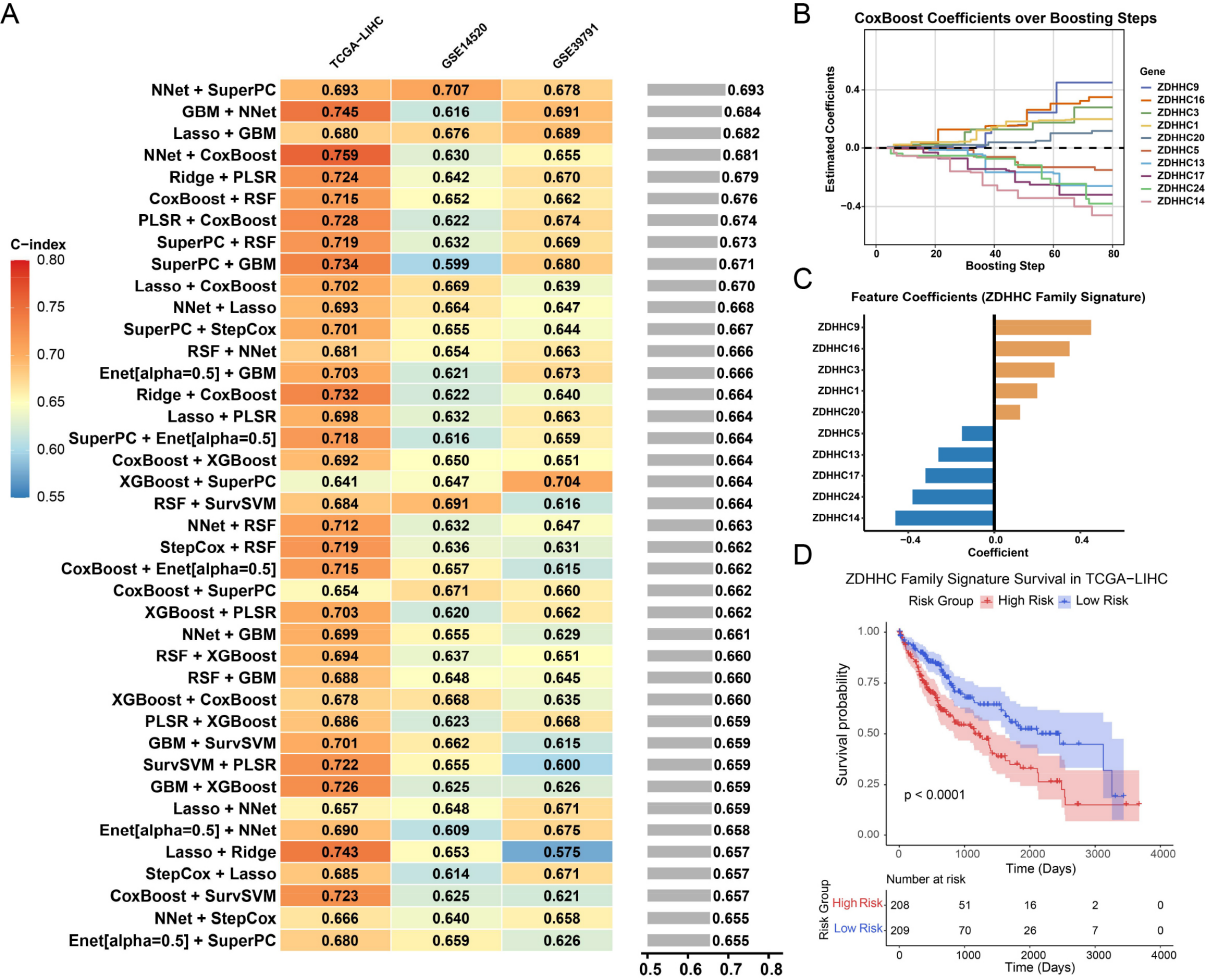
Construction and validation of a multi-gene ZDHHC prognostic signature using a consistent machine learning framework. **(A)** Evaluation of the predictive performance of ZDHHC family genes using a consistent machine learning framework. This framework paired Elastic Net-penalized Cox regression (Enet) with various algorithms (RSF, Lasso, CoxBoost, StepCox). The model framework with optimal generalizability was identified by calculating the concordance index (C-index) for each model pair across the TCGA-LIHC cohort and two independent GEO validation cohorts (GSE14520, GSE39791). **(B)** Dynamic process of feature selection by the CoxBoost algorithm. The heatmap shows the convergence trajectory of regression coefficients for ZDHHC family genes across increasing boosting steps. **(C)** The final optimal multi-gene prognostic signature. This signature includes pro-tumor/risk genes and protective genes. **(D)** Kaplan-Meier survival analysis based on the risk score derived from the multi-gene prognostic signature. Results show that patients in the high-risk group had significantly worse overall survival compared to the low-risk group in both the training and validation cohorts.

To construct a clinically robust prognostic model, we integrated the TCGA-LIHC cohort with two independent GEO microarray cohorts of liver cancer (GSE14520, GSE39791). We employed a “consistent machine learning framework” that paired Elastic Net penalized Cox regression (Enet) with various other algorithms (RSF, Lasso, CoxBoost, StepCox). The predictive performance of ZDHHC family genes was comprehensively evaluated by calculating the concordance index (C-index) for each model pair across all three cohorts (**Figure 1A**), thereby identifying the model framework with optimal generalizability.

During feature selection, the CoxBoost algorithm demonstrated the dynamic convergence trajectory of ZDHHC family gene coefficients across boosting steps (**Figure 1B**). Ultimately, we extracted an optimal multi-gene prognostic signature, which included ZDHHC9, ZDHHC16, ZDHHC3, ZDHHC1, and ZDHHC20 (pro-tumor/risk genes, coefficient > 0), as well as protective genes such as ZDHHC14 and ZDHHC24 (coefficient < 0) (**Figure 1C**). Based on the median expression level of each signature gene, patients were divided into high- and low-expression groups. Subsequently, Kaplan-Meier survival analysis consistently demonstrated that, according to the Cox model, high-risk group patients with liver cancer exhibited significantly worse survival outcomes compared to the low-risk group (**Figure 1D**). These results robustly confirm the powerful predictive value of this multi-gene family signature in the independent cross-cohort validation for liver cancer.

### 3.2 Expression profiling of the ZDHHC family in HCC

**Figure 2:**
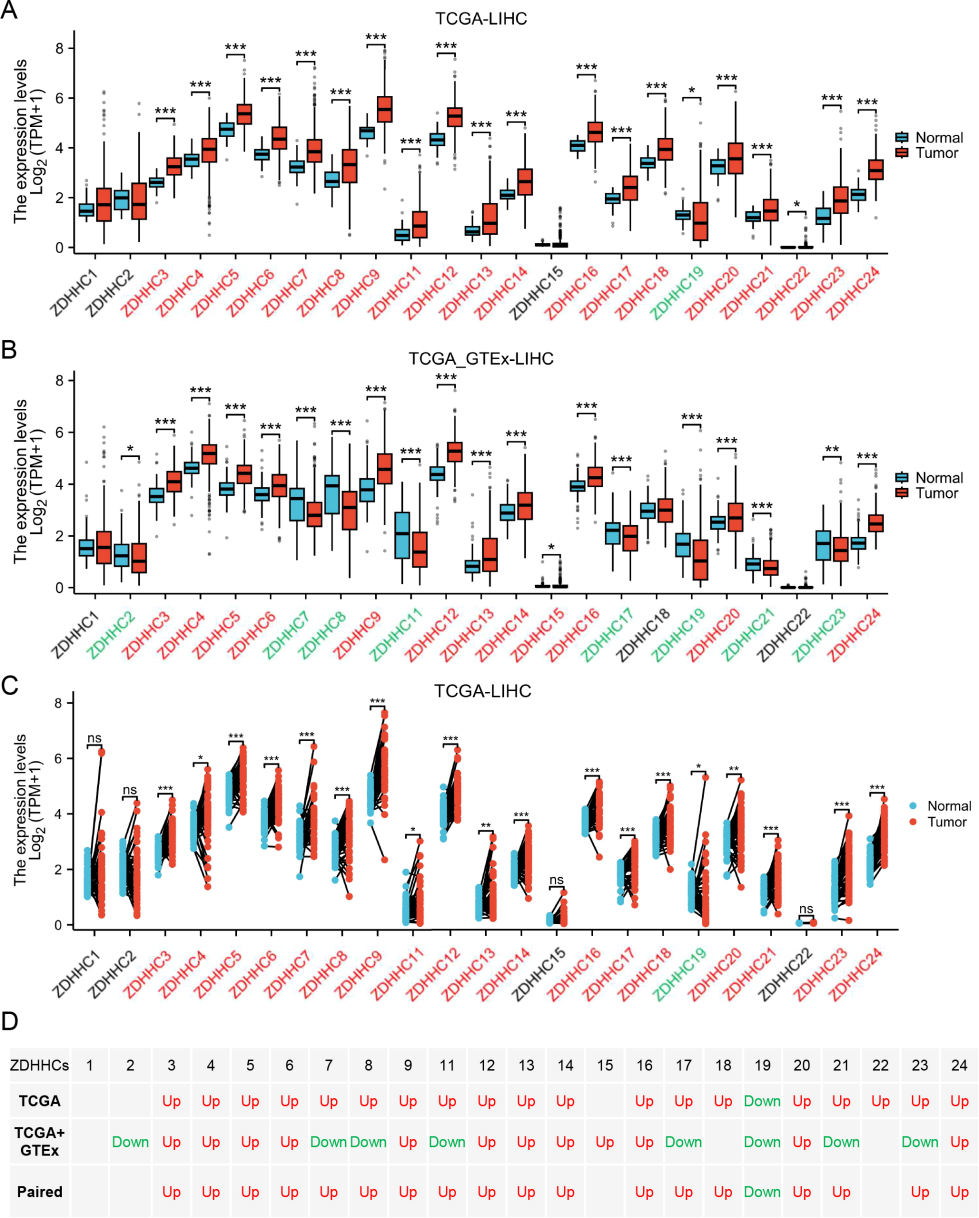
ZDHHC family mRNA expressions between HCC and normal tissues. **(A, B)** The ZDHHC family mRNA in HCC analysis via the TCGA/GTEx databases. **(C)** Expression of ZDHHC family members in HCC and paired adjacent normal tissues. **(D)** A consolidated overview of ZDHHC family mRNA in HCC as reported. ns, *P* ≥ 0.05; * *P* < 0.05; ** *P* < 0.01; *** *P* < 0.001. Group comparisons in **(A–C)** were performed using the Wilcoxon rank-sum test.

This study employed a multi-tiered analytical approach. Initially, we conducted a systematic evaluation of the ZDHHC family landscape in HCC to pinpoint core members with clinical relevance. Subsequently, we concentrated on the most promising candidate, ZDHHC9, for in-depth mechanistic and functional investigation within the HCC context. Finally, to assess its broader oncogenic significance, we expanded our analysis to characterize ZDHHC9 across a spectrum of human malignancies. Interrogation of TCGA-LIHC data revealed that 19 ZDHHC family members were upregulated in HCC specimens compared to normal tissues (**Figure 2A**). This finding was corroborated by an integrated analysis incorporating normal samples from the GTEx database, which confirmed elevated expression of 12 ZDHHC members in tumors (**Figure 2B**). Examination of paired tumor and adjacent non-tumor tissues within the TCGA-LIHC cohort further demonstrated that 12 ZDHHC genes were significantly overexpressed in malignant tissues (**Figure 2C**). These collective observations suggest that the ZDHHC family may exert a significant regulatory influence in liver cancer pathogenesis.

### 3.3 Prognostic significance of the ZDHHC family in HCC

**Figure 3:**
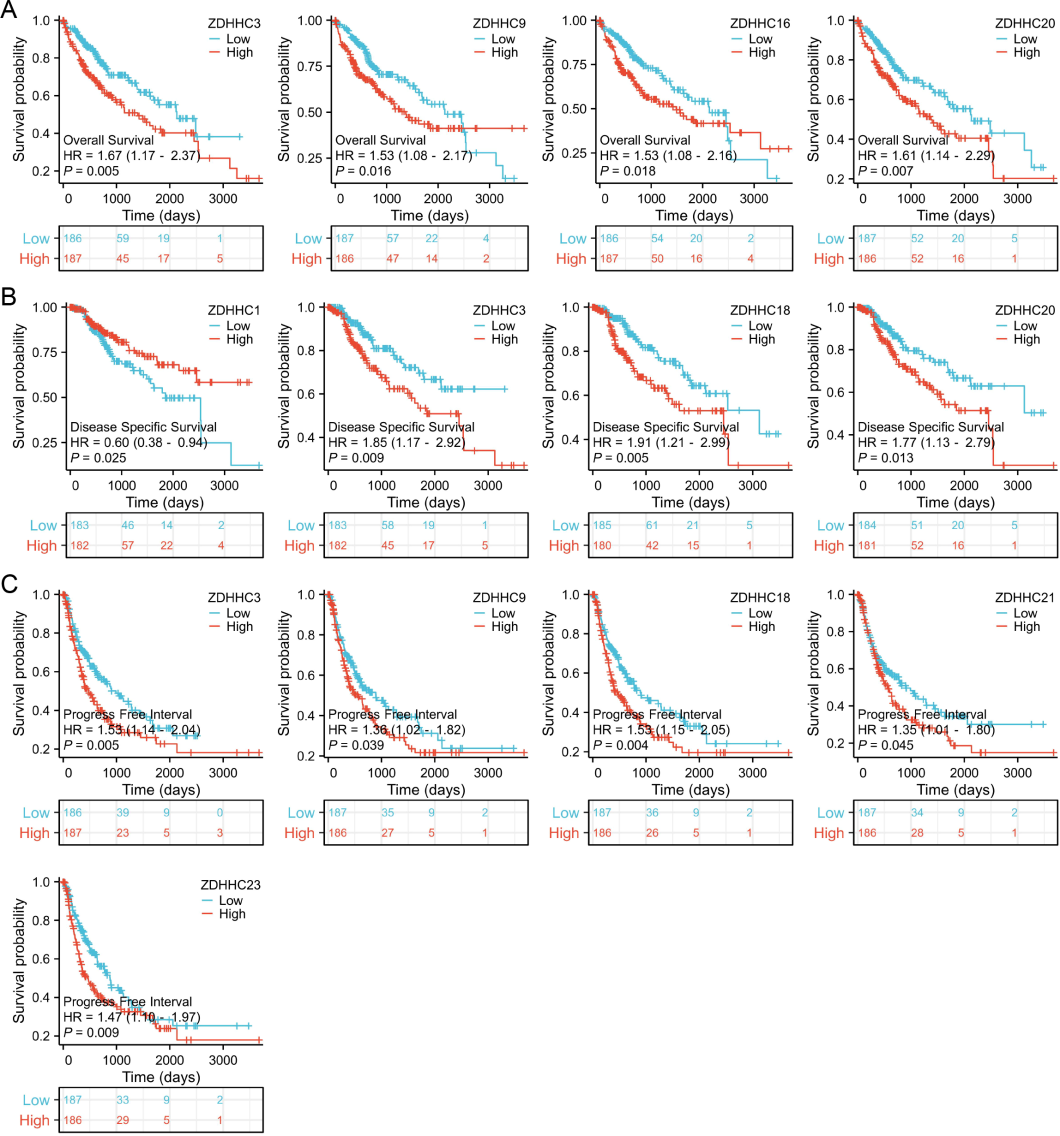
Prognostic analysis of ZDHHC family in HCC. **(A)** Relationship between ZDHHC3, ZDHHC9, ZDHHC16, and ZDHHC20 expression levels and OS in patients with HCC. **(B)** Relationship between ZDHHC1, ZDHHC3, ZDHHC18, and ZDHHC20 expression levels and DSS in patients with HCC. **(C)** Relationship between ZDHHC3, ZDHHC9, ZDHHC18, ZDHHC21, and ZDHHC23 expression levels and PFI in HCC. Statistical significance in **(A–C)** was determined by the log-rank test.

To elucidate the prognostic value of ZDHHC expression, we systematically evaluated the direct association between all 23 family members and key survival endpoints-overall survival (OS), disease-specific survival (DSS), and progression-free interval (PFI)-in HCC patients. Our analysis identified ZDHHC3, ZDHHC9, ZDHHC16, and ZDHHC20 as significantly correlated with OS (**Figure 3A**). For DSS, ZDHHC1, ZDHHC3, ZDHHC18, and ZDHHC20 showed significant associations (**Figure 3B**). Regarding PFI, ZDHHC3, ZDHHC9, ZDHHC18, and ZDHHC21 were found to be relevant (**Figure 3C**). These results indicate that multiple ZDHHC family members may serve as relatively specific prognostic biomarkers for HCC.

### 3.4 Mutational landscape of the ZDHHC family in HCC

**Figure 4:**
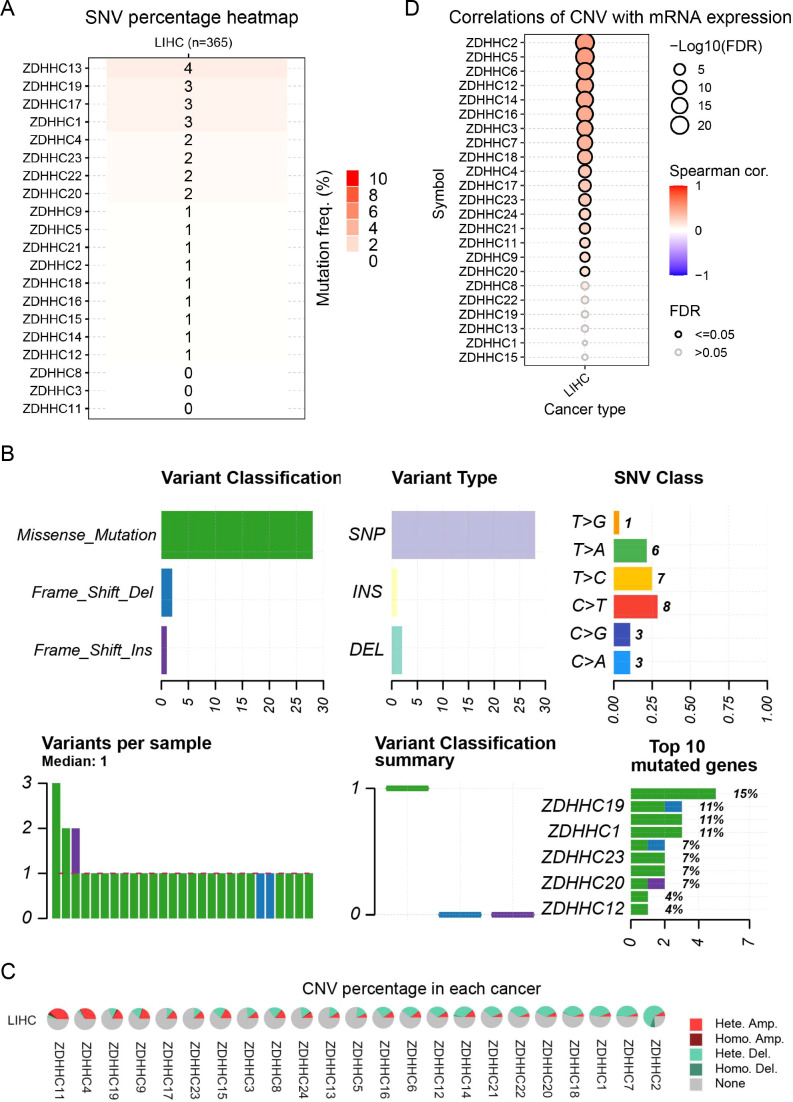
Mutation analysis of ZDHHC family in HCC. **(A)** The profile of SNV of the ZDHHC family genes in the HCC. **(B)** The SNV classes of ZDHHC family genes in the HCC. **(C)** The CNV of ZDHHC family genes in the HCC. **(D)** The correlations between CNV and ZDHHC family mRNA expression in the HCC.

Given that genetic alterations are a hallmark of cancer cells (31), we further investigated mutations within the ZDHHC family in HCC using the Gene Set Cancer Analysis (GSCA) platform (32). Analysis revealed that ZDHHC13 exhibited the highest frequency of single nucleotide variants (SNVs) among the family, whereas ZDHHC8, ZDHHC3, and ZDHHC11 showed no detectable SNVs (**Figure 4A**). Missense mutations constituted the predominant alteration type across the family (**Figure 4B**). Additionally, we assessed the copy number variation (CNV) status of these genes. The ZDHHC11 gene displayed the largest CNV percentage. Notably, the CNV of the ZDHHC2 gene showed a significant positive correlation with its mRNA expression level in HCC (**Figures 4C, D**).

### 3.5 Cross-cohort sc-RNA-seq reveals HCC microenvironment features and identifies ZDHHC9 as a core palmitoylation gene

**Figure 5:**
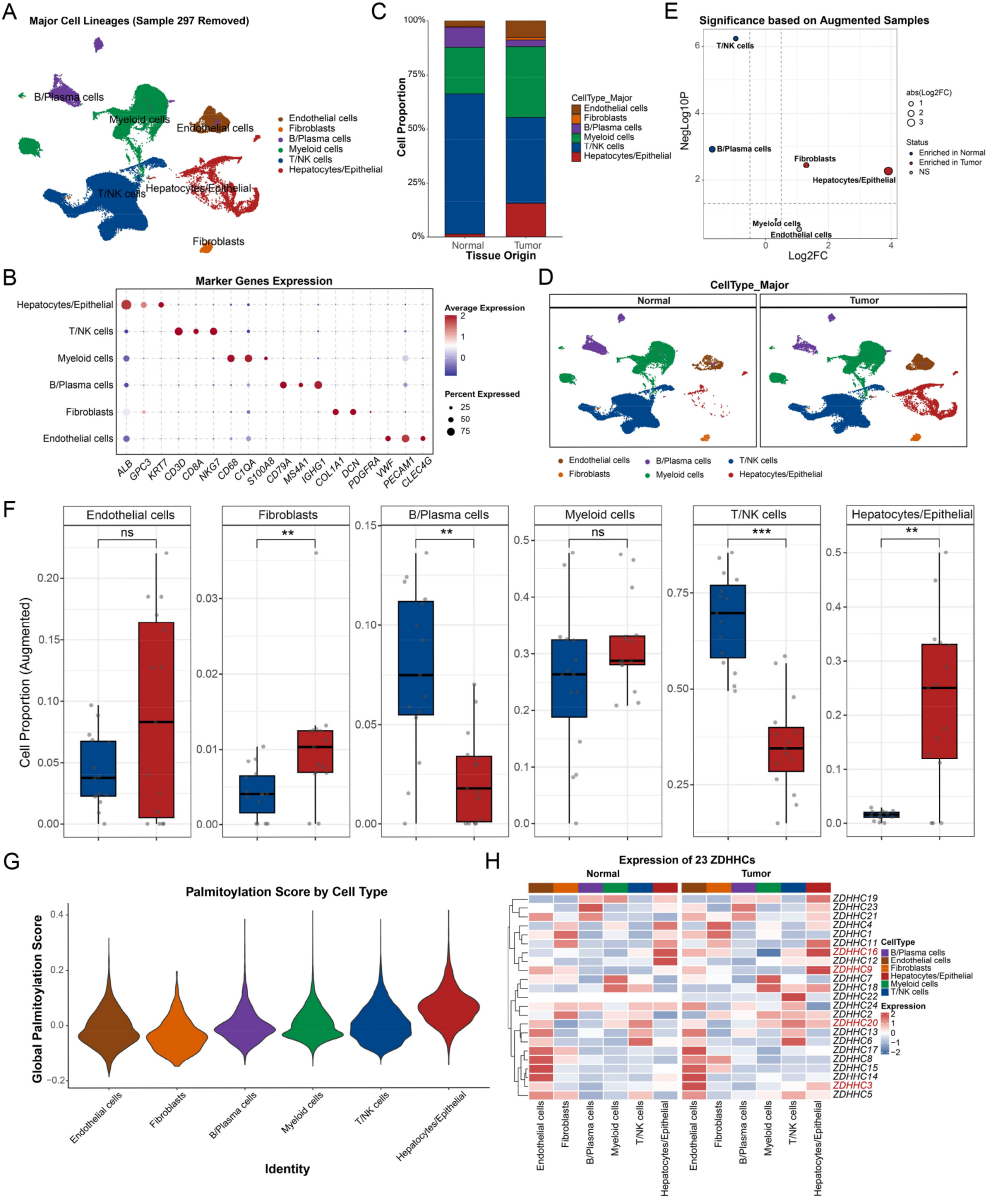
Integrated single-cell transcriptomic atlas reveals TME and ZDHHC9-mediated specific activation of palmitoylation in HCC. **(A)** Distribution of all cells in the UMAP space after integrated analysis of two independent HCC single-cell datasets (GSE299340 and GSE290298). Different colors represent the annotated major cell types. **(B)** Dot plot showing the expression of classic marker genes used to identify the six major cell clusters. The size of the dot indicates the percentage of cells expressing the gene, and the color intensity represents the average expression level. **(C)** Faceted UMAP plots separated by tissue origin (Normal vs. Tumor), displaying the distribution of major cell types. **(D)** Stacked bar chart showing the overall proportional composition of each major cell type in normal and tumor tissues. **(E)** Volcano plot of cellular proportion differences. Each point represents a cell type, with its position indicating the log2 fold change in proportion between tumor and normal tissues and the statistical significance. Red and blue dots denote cell types significantly enriched in tumor or normal tissues, respectively (Wilcoxon rank-sum test). **(F)** Box plots comparing the proportional distribution of each major cell type between normal and tumor tissues. (Wilcoxon rank-sum test); **(G)** Violin plots showing the distribution of the “Palmitoylation Score”, calculated based on the expression of the ZDHHC gene family, across different cell types. Hepatocytes/Malignant epithelial cells exhibited the highest median score. **(H)** Heatmap displaying the expression patterns (Z-score normalized) of all 23 ZDHHC family genes across different cell types. ZDHHC9 (indicated by a red arrow) shows specific high expression in hepatocytes/malignant epithelial cells from tumor tissues. ns, *P* ≥ 0.05, *P < 0.05, ** *P* < 0.01, *** *P* < 0.001.

To systematically characterize the TME of HCC and ensure the robustness of our findings, we downloaded and integrated two independent HCC single-cell RNA sequencing (scRNA-seq) datasets (GSE299340 and GSE290298) from the GEO database. Through integrated analysis of cells from paired tumor and adjacent normal tissues, followed by stringent quality control, dimensionality reduction, and clustering, we identified six major cell clusters in the UMAP projection (**Figure 5A**). Dot plots of classic marker genes (**Figure 5B**) annotated these clusters as Hepatocytes/Malignant Epithelial cells (high expression of ALB, GPC3), T/NK cells (high expression of CD3D, NKG7), Myeloid cells (high expression of CD68, C1QA), B/Plasma cells (high expression of CD79A, MS4A1), Fibroblasts (high expression of COL1A1, DCN), and Endothelial cells (high expression of VWF, PECAM1).

Comparative analysis of cellular composition between normal and tumor tissues, visualized by stacked bar charts and faceted UMAP plots (**Figures 5C, D**), revealed a substantial expansion of malignant epithelial cells within the TME. Enhanced statistical analysis based on data distribution further quantified this shift. A volcano plot of cellular proportion changes (**Figure 5E**) and statistical box plots (**Figure 5F**) collectively demonstrated significant TME remodeling in HCC: tumor tissues showed significant enrichment of malignant epithelial cells and fibroblasts, alongside marked depletion of T/NK cells and B/Plasma cells, confirming an immunosuppressive milieu.

We then assessed palmitoylation pathway activity at single-cell resolution. Violin plots of the “Palmitoylation Score”, calculated based on the expression of the ZDHHC gene family (**Figure 5G**), showed that Hepatocytes/Malignant Epithelial cells had the highest median score among all cell types, indicating this modification is primarily active within the tumor parenchyma. To pinpoint the core regulatory factors, we generated a comprehensive expression heatmap of all 23 ZDHHC genes (**Figure 5H**). Cross-cohort analysis identified several ZDHHC genes specifically upregulated in tumor epithelial cells. Among them, ZDHHC9 exhibited a particularly notable pattern: it was highly expressed in malignant epithelial cells from tumor tissues at levels significantly exceeding those in normal hepatocytes, while its expression was minimal in immune and stromal cells. This highly specific expression profile underscores the potential of ZDHHC9 as a promising therapeutic target in HCC.

### 3.6 Single-cell analysis reveals malignant epithelial subpopulations in HCC and implicates ZDHHC9 in hypoxic phenotype regulation

**Figure 6:**
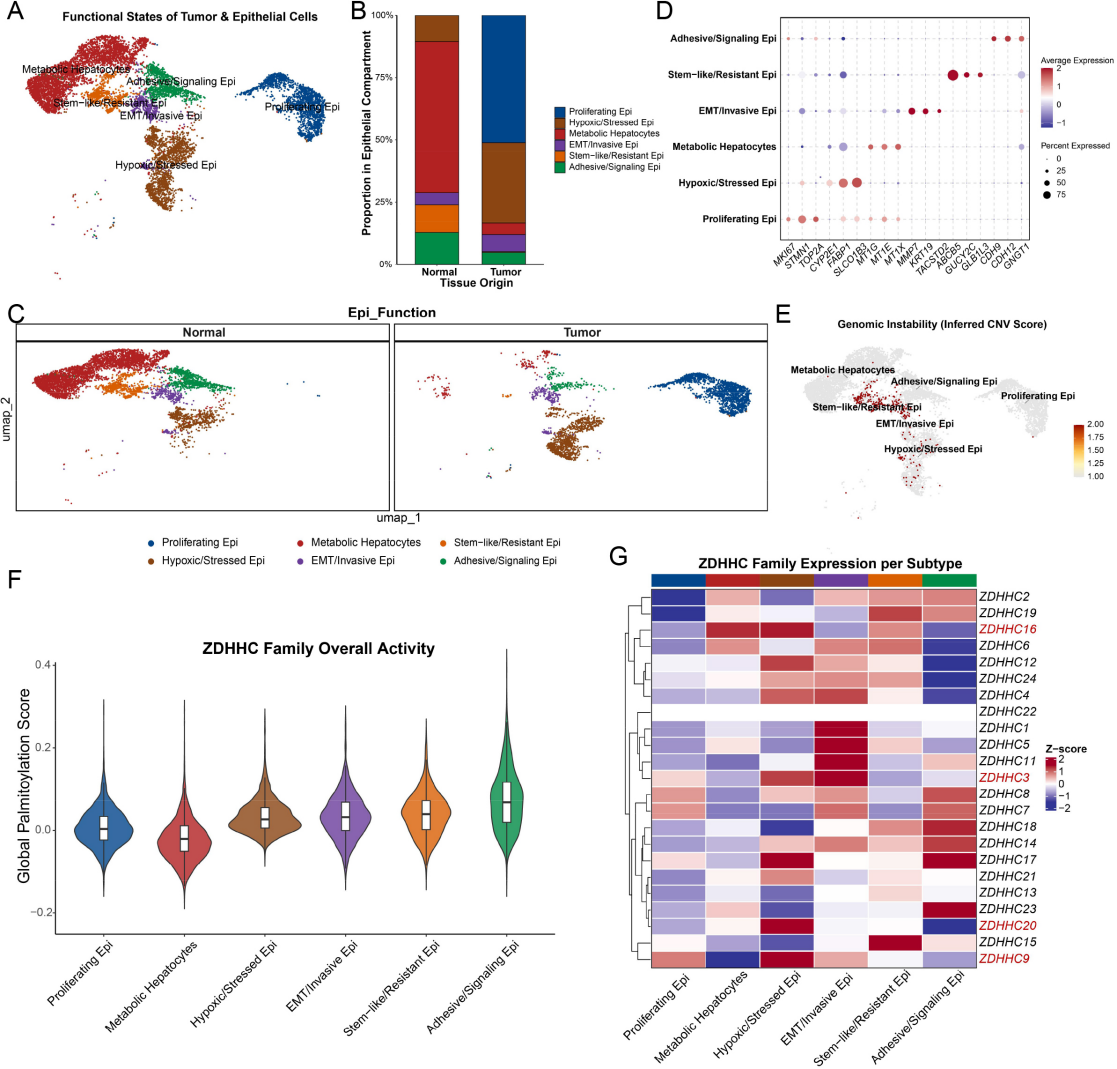
Sub-clustering of epithelial cells reveals diverse malignant states and highlights ZDHHC9 in driving a hypoxic/stressed phenotype. **(A)** UMAP plot showing six functionally distinct subpopulations identified from the hepatocyte/epithelial cell cluster. **(B)** Stacked bar chart displaying the proportional distribution of the six epithelial subpopulations in normal and tumor tissues. **(C)** UMAP plots of epithelial subpopulations, faceted by tissue origin (Normal vs. Tumor), highlighting the high enrichment of malignant phenotypes in tumor tissues. **(D)** Dot plot showing representative marker genes defining the specific biological functions of each epithelial subpopulation. **(E)** UMAP feature plot of the inferred-CNV score. High genomic instability clearly distinguishes bona fide malignant tumor cells (EMT/Invasive, Stem-like/Resistant, Proliferating subpopulations) from non-malignant normal hepatocytes. **(F)** Violin plots quantifying the global “Palmitoylation Score” across the six epithelial subpopulations. **(G)** Clustered heatmap displaying the expression profiles of ZDHHC family members across different epithelial subpopulations. The expression pattern of ZDHHC9 aligns with overall palmitoylation activity and shows prominent, specific high expression in the Hypoxic/Stressed subpopulation (indicated by a red arrow).

Given that epithelial cells constitute the tumor parenchyma of HCC, we further extracted all epithelial/hepatocyte clusters for unsupervised dimensionality reduction and subclustering analysis. In the UMAP space, epithelial cells were finely partitioned into six subpopulations with distinct functional states (**Figure 6A**). By analyzing their tissue origin (**Figures 6B, C**) and specific marker genes (**Figure 6D**), we found these subpopulations exhibited markedly different distribution patterns and functional characteristics: subpopulations enriched in normal tissues primarily consisted of classical Metabolic Hepatocytes (high expression of CYP2E1, FABP1) and Adhesive/Signaling Epithelial cells. In contrast, populations enriched in tumor tissues displayed overt malignant features, including Proliferating Epithelial cells (high expression of MKI67, TOP2A), Hypoxic/Stressed Epithelial cells (high expression of MT1G, MT1E), EMT/Invasive Epithelial cells (high expression of MMP7, KRT19), and Stem-like/Resistant Epithelial cells (high expression of ABCG2).

To confirm the malignant nature of these cells at the genomic level, we inferred CNV burden from the single-cell transcriptomic data. The results (**Figure 6E**) showed that high genomic instability (high CNV score) was precisely enriched in the tumor-derived Proliferating, Hypoxic, EMT/Invasive, and Stem-like subpopulations, whereas Metabolic Hepatocytes from normal tissues exhibited minimal CNV scores. This conclusively verified that these four tumor-enriched subpopulations represent the bona fide malignant parenchymal cells of HCC.

To delve into the role of palmitoylation in HCC malignant progression, we evaluated the “Palmitoylation Score” across these six epithelial subpopulations. Violin plot analysis revealed (**Figure 6F**) that, compared to normal Metabolic Hepatocytes, the palmitoylation pathway was aberrantly and significantly activated in the highly invasive EMT/Invasive subpopulation, the therapy-resistant Stem-like/Resistant subpopulation, and the Hypoxic/Stressed subpopulation.

To identify the core enzyme driving this process, we systematically examined the expression profiles of ZDHHC family members across different subpopulations (**Figure 6G**). The clustered heatmap indicated that different ZDHHC genes played heterogeneous roles in maintaining distinct cellular states. Notably, the expression pattern of ZDHHC9 closely aligned with the overall palmitoylation activity, showing the most prominent and specific high expression in the Hypoxic/Stressed subpopulation. This key finding strongly suggests that ZDHHC9 may function as a central palmitoyltransferase, potentially driving HCC cells toward a hypoxic and stressed phenotype by remodeling the lipid modification network of its target proteins.

### 3.7 Immune infiltration analysis of ZDHHC9/3/16/20 in HCC

**Figure 7:**
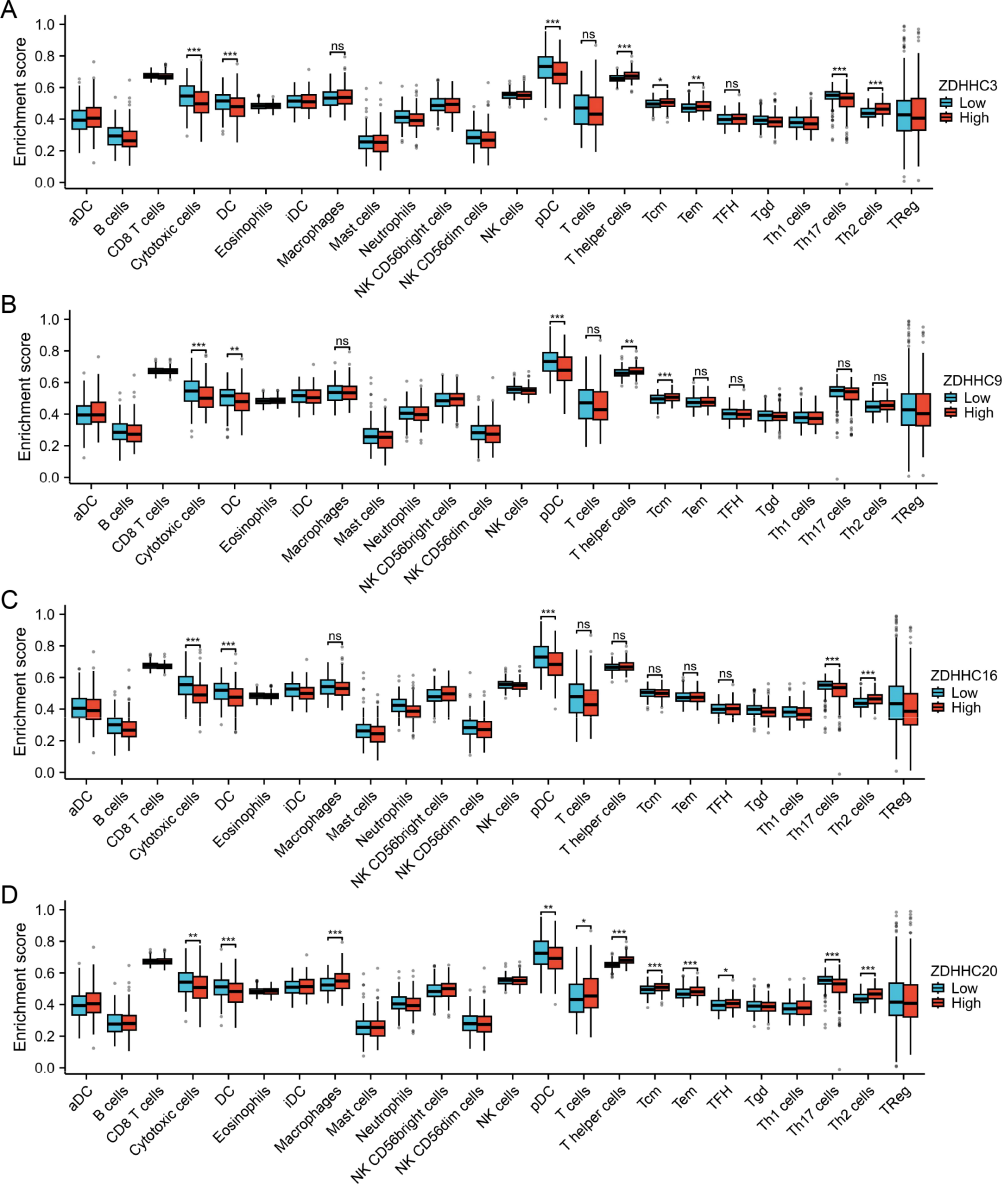
Assessment of immune infiltration. **(A–D)** Analysis of immune infiltration differences between the ZDHHC3, ZDHHC9, ZDHHC16, and ZDHHC20-high and ow groups using ssGSEA in TCGA. ns, *P* ≥ 0.05; * *P* < 0.05; ** *P* < 0.01; *** *P* < 0.001. The Wilcoxon rank-sum test was applied for all comparisons, as the immune cell scores were not normally distributed.

Considering the heterogeneity of the HCC tumor microenvironment, we performed an immune infiltration analysis on the four prognostically relevant ZDHHC members (ZDHHC3, ZDHHC9, ZDHHC16, and ZDHHC20) using TCGA data. Single-sample gene set enrichment analysis (ssGSEA) indicated that high expression groups of ZDHHC3, ZDHHC9, ZDHHC16, and ZDHHC20 were associated with lower infiltration scores for activated cytotoxic cells, dendritic cells (DCs), and plasmacytoid dendritic cells (pDCs), respectively (**Figures 7A–D**). Dendritic cells are pivotal antigen-presenting cells within the immune system, capable of recognizing tumor antigens and activating T-cell-mediated anti-tumor responses. Therapeutic strategies targeting DCs, in combination with adoptive T-cell therapy, hold promise as adjuvant treatments for HCC. Our findings suggest that ZDHHC3, ZDHHC9, ZDHHC16, and ZDHHC20 may facilitate HCC progression by modulating immune cell infiltration, potentially contributing to an immunosuppressive microenvironment and immune escape.

### 3.8 Cancer drug sensitivity analysis of ZDHHC3/9/16/20

To investigate the association between ZDHHC genes and therapeutic response, we performed drug resistance analyses via the GSCA platform (32). The results indicated that expression levels of all four genes were positively correlated with the half-maximal inhibitory concentration (IC50) of the majority of tested compounds. Among these, ZDHHC9 exhibited the strongest positive association with drug resistance (**Supplementary Figures 1A, B**).

### 3.9 Machine learning identifies characteristic genes

**Figure 8:**
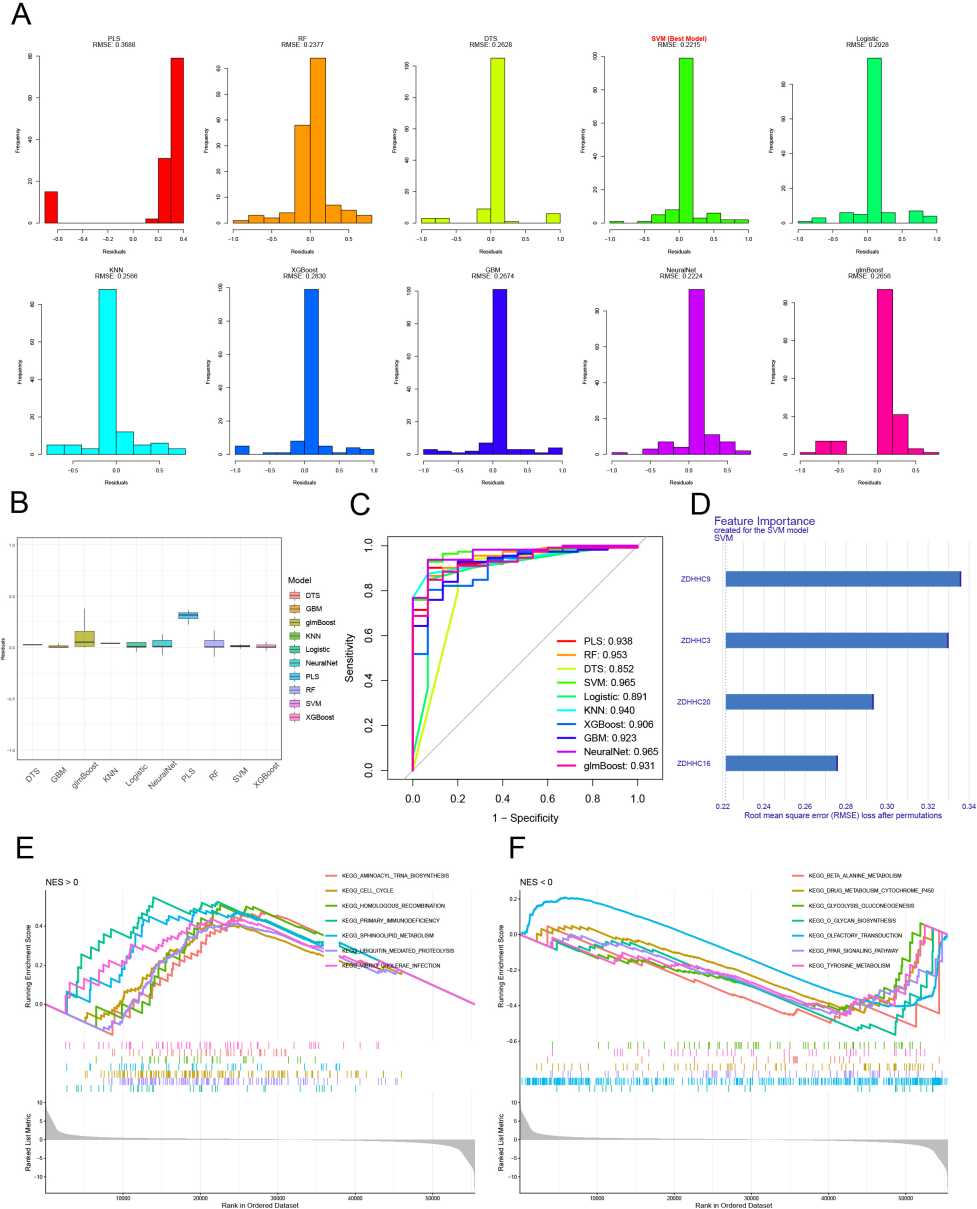
Machine learning identifies signature genes. **(A)** Histogram of residual error distribution of 10 machine learning models. **(B)** Box plots of residual of the 10 machine learning models. **(C)** Receiver operating characteristic curves of ten machine learning models. **(D)** Bar graph of gene importance score of SVM machine learning model. **(E)** Enrichment map of gene set enrichment analysis (NES > 0, *P* < 0.05, FDR < 0.25). **(F)** Enrichment map of gene set enrichment analysis (NES < 0, *P* < 0.05, FDR < 0.25).

Given the marked upregulation of ZDHHC3, ZDHHC16, ZDHHC20, ZDHHC9, and ZDHHC21 in tumor tissues and their significant links to patient prognosis, we constructed ten distinct machine learning models to identify diagnostically valuable signature genes. Comparative evaluation revealed that the support vector machine (SVM) algorithm demonstrated superior diagnostic performance, achieving the highest area under the curve (AUC = 0.965) and the smallest residual mean square error (RMSE = 0.2215). Utilizing this model for feature importance scoring identified ZDHHC9 as the top-ranked gene, highlighting its exceptional potential for application in cancer diagnosis (**Figures 8A–D**).

Subsequent gene set enrichment analysis (GSEA) was performed. Pathways significantly enriched in the ZDHHC9 high-expression group included Necroptosis (FDR = 0.140, P = 0.0008), Protein processing in endoplasmic reticulum (FDR = 0.160, P = 0.001), Dilated cardiomyopathy (FDR = 0.169, P = 0.001), Hypertrophic cardiomyopathy (FDR = 0.169, P = 0.001), Aldosterone synthesis and secretion (FDR = 0.170, P = 0.001), Adrenergic signaling in cardiomyocytes (FDR = 0.176, P = 0.001), and Renin secretion (FDR = 0.183, P = 0.002), suggesting potential involvement in cell death regulation during tumor progression. Conversely, pathways enriched in the ZDHHC9 low-expression group included Oxidative phosphorylation (FDR = 0.152, P = 0.0009), Thermogenesis (FDR = 0.161, P = 0.001), Parkinson disease (FDR = 0.168, P = 0.001), African trypanosomiasis (FDR = 0.169, P = 0.001), Prion disease (FDR = 0.169, P = 0.001), Chemical carcinogenesis-reactive oxygen species (FDR = 0.170, P = 0.001), and Huntington disease (FDR = 0.170, P = 0.001), hinting at potential disruptions in cellular energy metabolism upon ZDHHC9 deficiency (**Figures 8E, F**).

These findings established ZDHHC9 as a central player in HCC. To determine whether its oncogenic role was specific to HCC or represented a broader phenomenon, we next conducted a comprehensive pan-cancer analysis.

### 3.10 Pan-cancer characterization reveals broad oncogenic relevance of ZDHHC9

We performed an integrated, multi-dimensional pan-cancer analysis to evaluate the oncogenic potential of ZDHHC9 beyond HCC. Analysis of TCGA data showed significant upregulation of ZDHHC9 transcript levels in tumor versus adjacent normal tissues across 14 cancer types (**Supplementary Figure 2A**). Integration with GTEx data further identified ZDHHC9 overexpression in 22 malignancies (**Supplementary Figure 2B**). Examination of paired TCGA samples confirmed elevated ZDHHC9 expression in 14 cancer types (**Supplementary Figure 2C**), indicating widespread dysregulation across human cancers.

Survival analysis associated elevated ZDHHC9 expression with poorer outcomes, including shorter overall survival in several tumors such as HCC (**Supplementary Figures 3A–D**), supporting its potential as a cross-cancer prognostic marker.

Interrogation of the genomic landscape using cBioPortal revealed the highest mutation frequencies for ZDHHC9 in endometrial carcinoma, melanoma, and renal clear cell carcinoma (**Supplementary Figure 4A**). Furthermore, copy number variations (CNVs) of ZDHHC9 were found to modulate its mRNA expression levels in various cancers (**Supplementary Figures 4B, C**). Analysis of DNA methylation identified significant differences in ZDHHC9 methylation levels between tumor and normal tissues in 6 cancer types (**Supplementary Figures 5A, B**), suggesting that both genetic and epigenetic mechanisms underlie its dysregulation.

Investigation using the TIMER database revealed significant associations between ZDHHC9 expression and the abundance of various tumor-infiltrating immune cell types across 30 cancer types. Spearman correlation analysis employing multiple algorithms consistently demonstrated significant correlations between ZDHHC9 expression and immune cell infiltration levels in the pan-cancer context (**Supplementary Figures 6A, B**), underscoring a strong link between ZDHHC9 and the tumor immune landscape.

Analysis of drug sensitivity data indicated that ZDHHC9 expression positively correlated with sensitivity to several chemotherapeutic agents, including clofarabine, doxorubicin, vinblastine, vorinostat, and etoposide (**Supplementary Figures 7A, B**), suggesting a potential role in predicting therapeutic response.

### 3.11 Spatial transcriptomics analysis of ZDHHC9 expression in HCC

**Figure 9:**
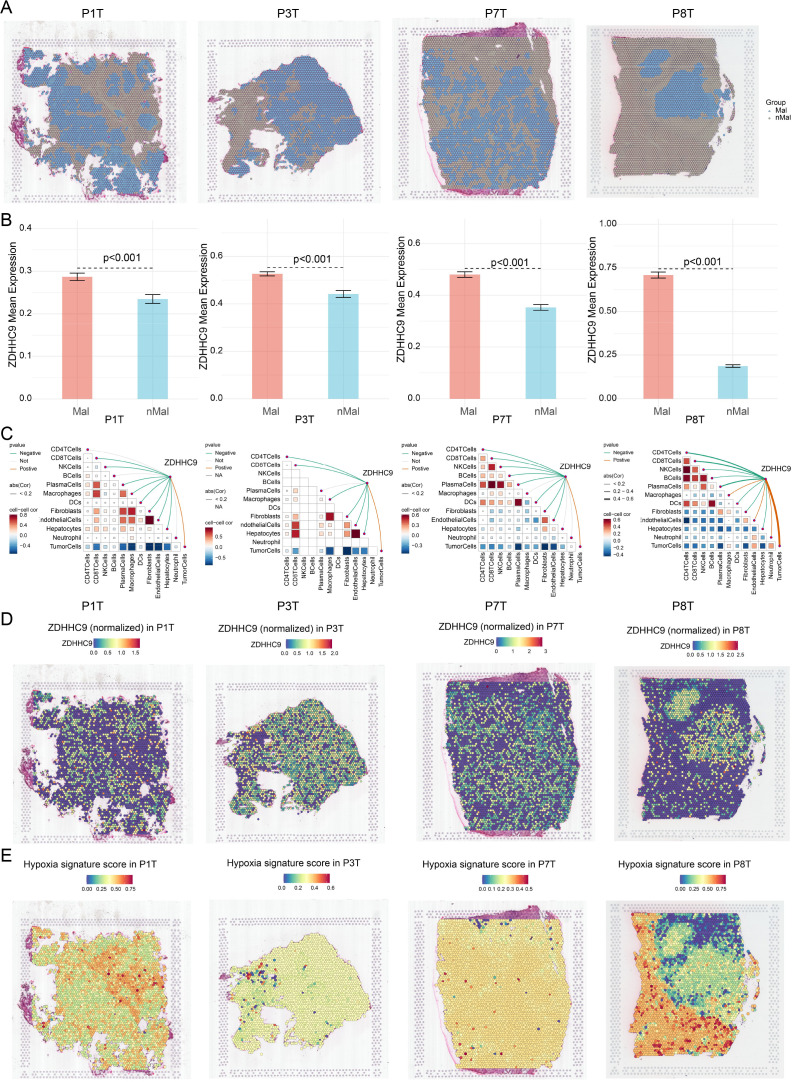
Spatial transcriptome analysis of ZDHHC9 expression characteristics in HCC. **(A)** Spatial arrangement features of malignant (Mal) and non-malignant (nMal) areas within individual spots. **(B)** Histogram depicting ZDHHC9 expression levels within both Mal and nMal-defined regions. **(C)** Spearman correlation analysis between ZDHHC9 gene expression and cell content in HCC spot. **(D)** Spatial distribution of ZDHHC9 gene expression across a tumor tissue section. **(E)** A hypoxia signature score was calculated for each spatial spot based on the expression levels of hypoxia-associated genes (FABP1, SLCO1B3, CYP2E1). The color map (from blue to red) represents the relative level of the hypoxia signature score, with red regions indicating areas with prominent hypoxic features.

We next examined the spatial expression profile of ZDHHC9 in HCC using published spatial transcriptomic data (28). Analysis of seven tissue sections via the Sparkle database enabled high-resolution microenvironment characterization. Visualization using the Spatial Feature Plot function revealed a striking spatial concordance between ZDHHC9 expression and regions rich in malignant cells (**Figures 9A, B**). ZDHHC9 expression levels exhibited a strong positive correlation with the proportion of tumor cells per spatial spot, consistent with prior cellular localization analyses (**Figure 9C**).

To directly validate whether ZDHHC9 spatially co-localizes with the hypoxic microenvironment, we performed a spatial co-localization analysis. We constructed a hypoxia signature score based on representative genes from the HIF-1α signaling pathway (FABP1, SLCO1B3, CYP2E1) and quantified the score for each spatial spot. As shown in **Figure 9D**, ZDHHC9 expression exhibited a focal, high-expression pattern in space (red regions). Superimposing these regions with the high hypoxia signature score regions (red) in **Figure 9E** revealed a high degree of spatial congruence. This finding intuitively demonstrates a tight spatial coupling between high ZDHHC9 expression and the local hypoxic microenvironment, providing strong spatial-level visualization evidence supporting the hypothesis that ZDHHC9 is centrally positioned in the hypoxia-immunosuppressive axis.

### 3.12 Silencing ZDHHC9 attenuates the aggressive phenotype of HCC

**Figure 10:**
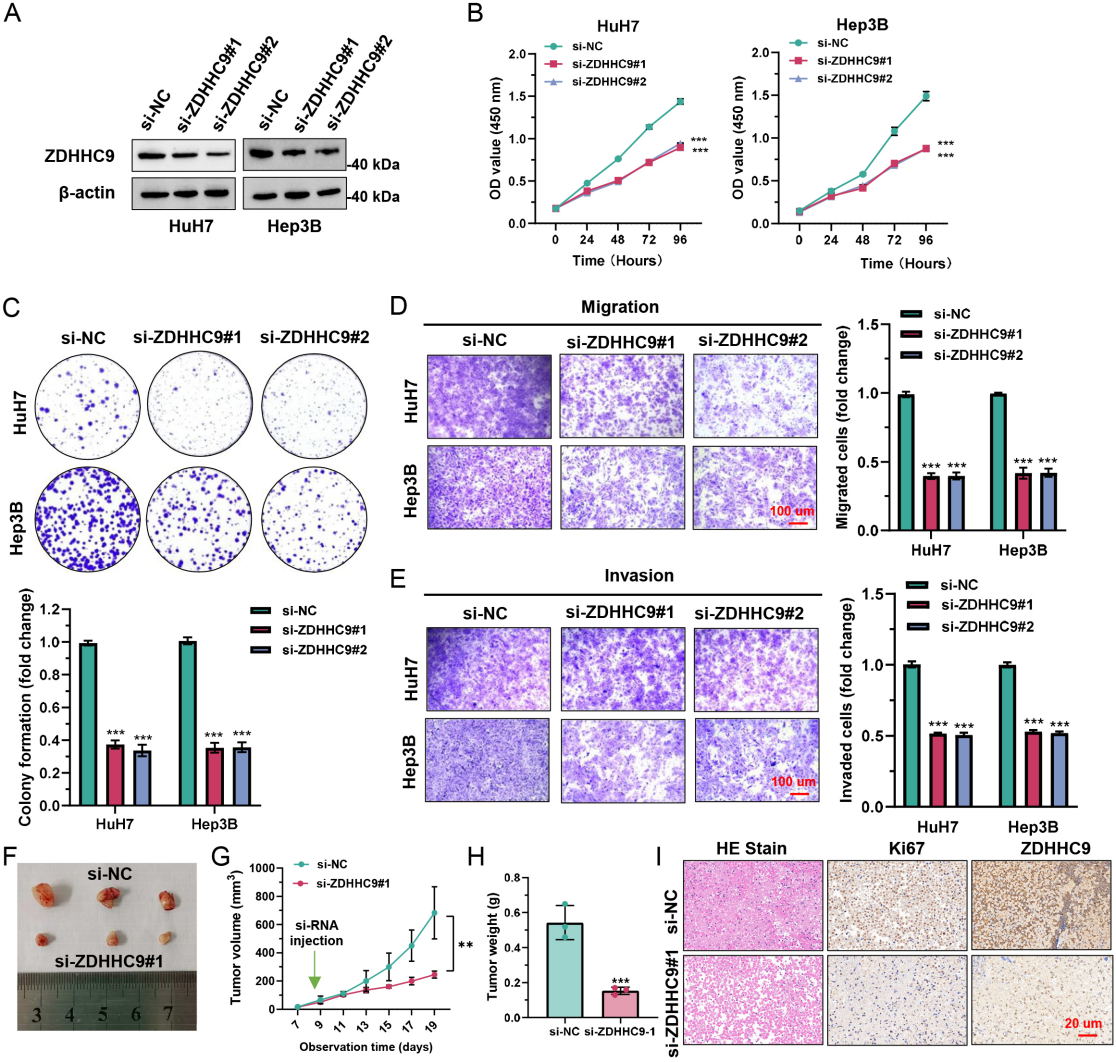
ZDHHC9 regulates HCC progression. **(A)** Western blot showed the knockdown effect of si-ZDHHC9 in HuH7 and Hep3B cells. **(B, C)** Effects of ZDHHC9 knockdown on the growth of HCC cells using CCK8 **(B)**, and colony formation **(C)** assays. **(D–E)** The transwell assay was used to explore the influence of ZDHHC9 on the metastasis of HCC cells. Scale bars: 100 µm. **(F)** Representative images of excised tumors. **(G)** Tumor volume growth curves over time in mice injected with control or ZDHHC9-knockdown HCC cells. **(H)** End-point tumor weights in the same xenograft model. **(I)** Representative immunohistochemical images and quantitative analysis of Ki67-positive cells in xenograft tumor sections. Scale bars: 20 µm. Data are presented as mean ± SD; ** P <0.01, *** P <0.001. Statistical significance was determined by two-tailed Student’s t-test, as the data met assumptions of normality and equal variance.

To elucidate the biological function of ZDHHC9 in HCC, we transfected Hep3B and Huh7 cells with siRNA targeting ZDHHC9. Western blot analysis confirmed a significant reduction in ZDHHC9 protein levels following transfection (**Figure 10A**). CCK-8 and colony formation assays demonstrated that ZDHHC9 knockdown significantly inhibited HCC cell proliferation (**Figures 10B, C**). Furthermore, Transwell assay results showed that ZDHHC9 silencing impeded the migration and invasive capacity of HCC cells (**Figures 10D, E**).

An *in vivo* xenograft model was employed to validate these findings. Compared to the control group, tumor growth was significantly suppressed in the ZDHHC9-knockdown group over 19 days, as evidenced by slower increases in tumor volume and reduced final tumor weights (**Figures 10F–H**). Immunohistochemical analysis further revealed lower positivity for the proliferation marker Ki67 in tumors with depleted ZDHHC9 expression (**Figure 10I**).

## 4 Discussion

HCC continues to be a leading contributor to cancer-related deaths worldwide (33). Protein S-palmitoylation is increasingly recognized as a crucial regulator of oncogenic pathways (34), therapy resistance, and immune modulation. Our comprehensive characterization of the ZDHHC family in HCC reveals a previously underappreciated role for this modification in disease progression, particularly through a “Hypoxia-ZDHHC9-Immunosuppression” axis.

Our finding that a subset of ZDHHC members (3, 9, 16, and 20) constitutes a robust prognostic module is consistent with recent reports implicating ZDHHC3 in HCC immune evasion via cholesterol metabolism (17) and ZDHHC20 in tumor development (16). However, our pan-cancer analysis extends this observation by suggesting that ZDHHC9, not previously highlighted in HCC, may act as a more central hub. While studies in other cancers have linked ZDHHC9 to proliferation (13), our work uniquely positions it at the intersection of hypoxia and immunity, a connection not yet reported. The strong predictive performance of our multi-gene signature, built using a consensus machine learning framework (35–38), further supports the clinical relevance of this enzyme family, aligning with methodological best practices for reducing overfitting.

The specific enrichment of ZDHHC9 in malignant epithelial cells under hypoxic stress, confirmed by single-cell and spatial transcriptomics, directly supports the proposed axis. This tumor-cell-specific expression pattern is a key finding, suggesting a potential therapeutic window. Direct spatial co-localization of ZDHHC9 with a hypoxia signature provides compelling evidence for its role in microenvironmental remodeling. This is broadly consistent with the known role of palmitoylation in stabilizing HIFs, but our data are the first to show this specific co-localization in the HCC TME. The observed correlation of ZDHHC9 high expression with an immunosuppressive microenvironment (reduced cytotoxic T cells and dendritic cells) aligns with emerging evidence linking palmitoylation to immune checkpoint regulation (31, 39). The top diagnostic importance score for ZDHHC9 in our machine learning models, along with its correlation with drug sensitivity, further strengthens its dual potential as both a biomarker and a therapeutic target. While the functional validation (silencing ZDHHC9 inhibits proliferation, migration, and invasion) is consistent with its pro-oncogenic role, the precise mechanisms by which it remodels the immune microenvironment warrant further investigation.

A pivotal extension of our work is the demonstration of ZDHHC9’s pan-cancer relevance. Its frequent upregulation and consistent association with poor prognosis across multiple cancer types (13, 40–43) suggests a conserved, microenvironment-driven mechanism. We propose that ZDHHC9 acts as a conserved hub, potentially palmitoylating a common set of substrate proteins involved in both hypoxic adaptation (HIF-1α stabilization) and immune evasion (PD-L1 upregulation). This hypothesis is supported by our pan-cancer immune correlation analysis (39, 43, 44). The diversity of its dysregulation mechanisms—from mutations and CNVs to epigenetic alterations—mirrors the complexity seen in other key oncogenic drivers (31) and suggests that multiple pathways converge on disrupting palmitoylation homeostasis in cancer.

Several aspects of our findings warrant nuanced comparison with existing literature. For instance, while ZDHHC9 is overexpressed across cancers, the specific downstream effects might be context-dependent due to tumor heterogeneity. The difference in the dominant mechanism of dysregulation between ZDHHC13 (SNVs) and ZDHHC11 (CNVs) highlights the need for cancer-type-specific analyses. Furthermore, our limitation—the reliance on transcriptomic data—is a recognized challenge in the palmitoylation field. Since enzyme activity is post-translationally regulated, elevated mRNA does not guarantee enhanced function. This disconnects between transcript and protein activity could explain why some studies might fail to find a functional role for ZDHHC9 in specific contexts. Future work using palmitoyl-proteomics is essential to bridge this gap and precisely map its downstream signaling networks.

In summary, our findings establish ZDHHC9 as a key mediator of a hypoxia-driven, immunosuppressive axis in HCC, with potential pan-cancer relevance. The combination of its tumor-cell-enriched expression, its role as a druggable enzyme with a well-defined active site, and its robust correlation with immunotherapy response markers positions it as a uniquely compelling therapeutic target. Future studies should focus on validating this axis in pre-clinical models using selective inhibitors, assessing its biomarker potential in prospective cohorts, and exploring its role in combination immunotherapy strategies. These directions are essential to translate our findings into clinical benefit.

## 5 Conclusions

Our work establishes that the ZDHHC family plays a significant regulatory role in HCC. Through integrated multi-omics analysis, we developed a robust multi-gene prognostic signature using a consistent machine learning framework, which demonstrated powerful predictive value across independent cohorts. We further identify ZDHHC9 as a core palmitoyl transferase with tumor cell-specific overexpression in HCC, particularly linked to a hypoxic phenotype and an immunosuppressive microenvironment. It serves as a novel diagnostic biomarker, a prognostic indicator, and a promising therapeutic target for HCC. Furthermore, extensive pan-cancer analysis reveals that ZDHHC9 exhibits broad dysregulation and clinical relevance across multiple cancer types, highlighting its potential as both a pan-cancer biomarker and a candidate for targeted therapy.

## Data Availability

The original contributions presented in the study are included in the article/supplementary material. Further inquiries can be directed to the corresponding authors.
