# Supplementary figures and images for: Multi-omics and spatial transcriptomics decode the ZDHHC9-driven hypoxia-immunosuppressive axis in hepatocellular carcinoma
Source: Front Oncol. 2026 Jun 17;16:1869712. doi: 10.3389/fonc.2026.1869712 (PMC13318649; doi:10.3389/fonc.2026.1869712)

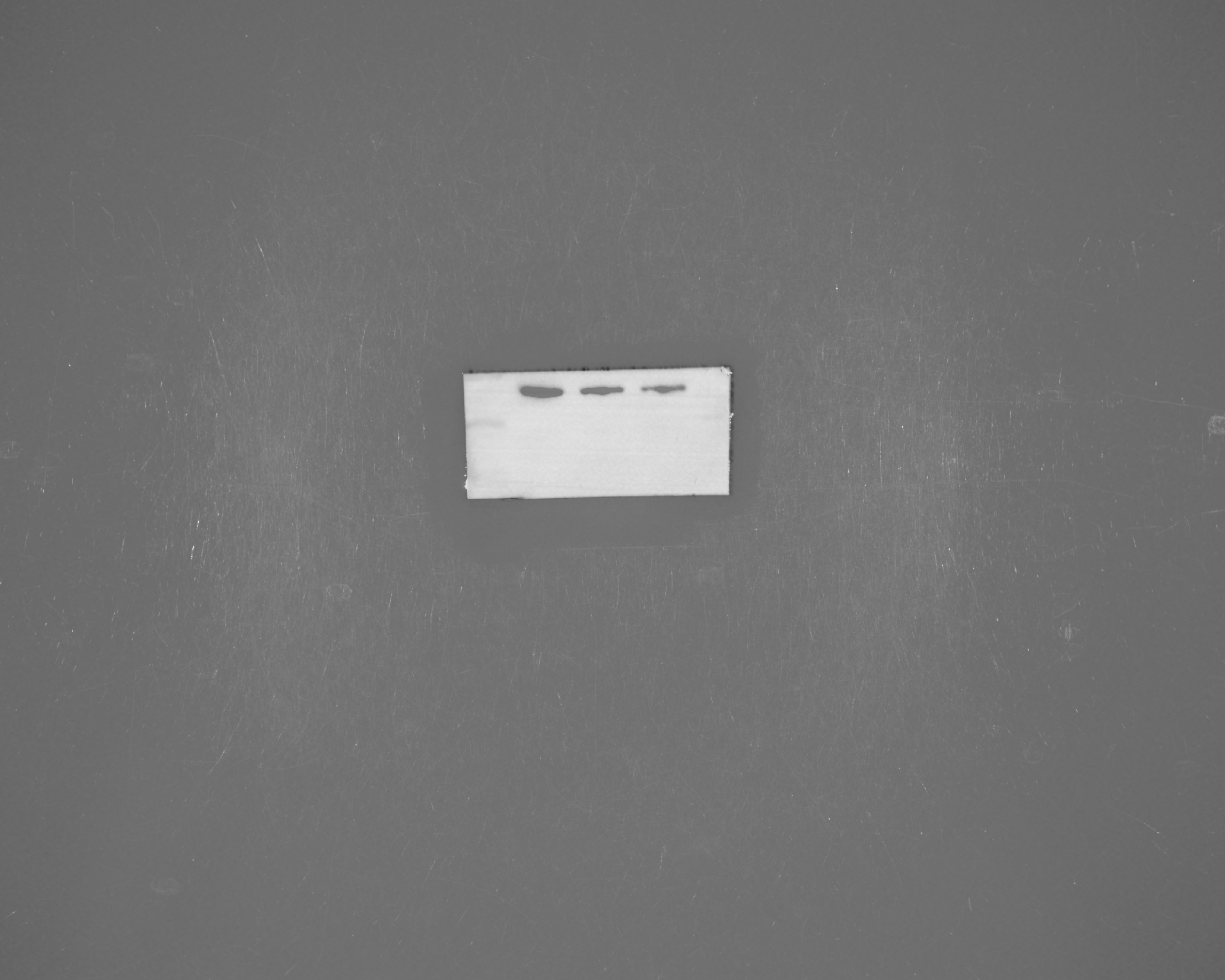

Supplement: Supplementary file 1 [file DataSheet1.zip › WB/Hep3B ZDHHC9.tif]

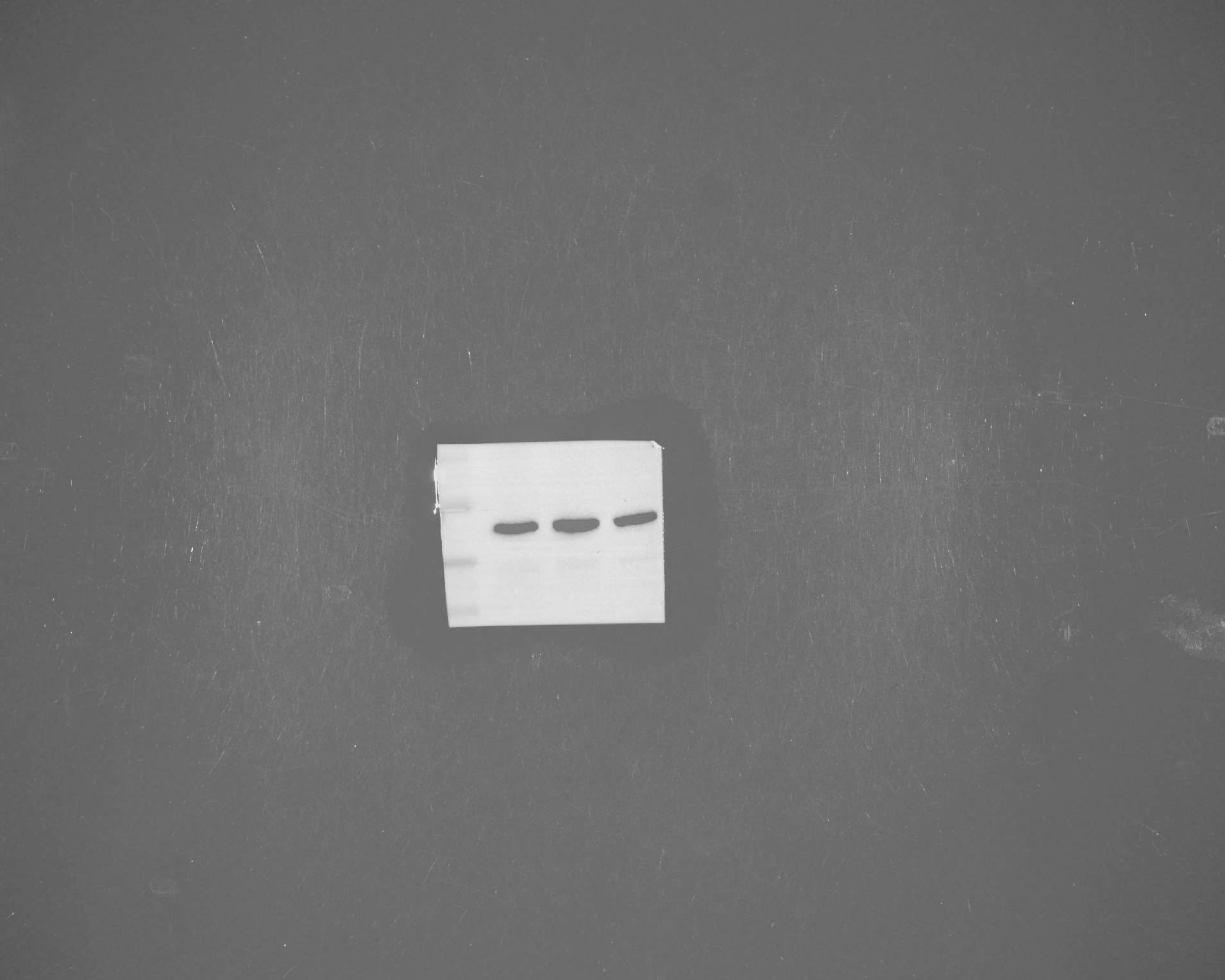

Supplement: Supplementary file 1 [file DataSheet1.zip › WB/Hep3B b-actin.tif]

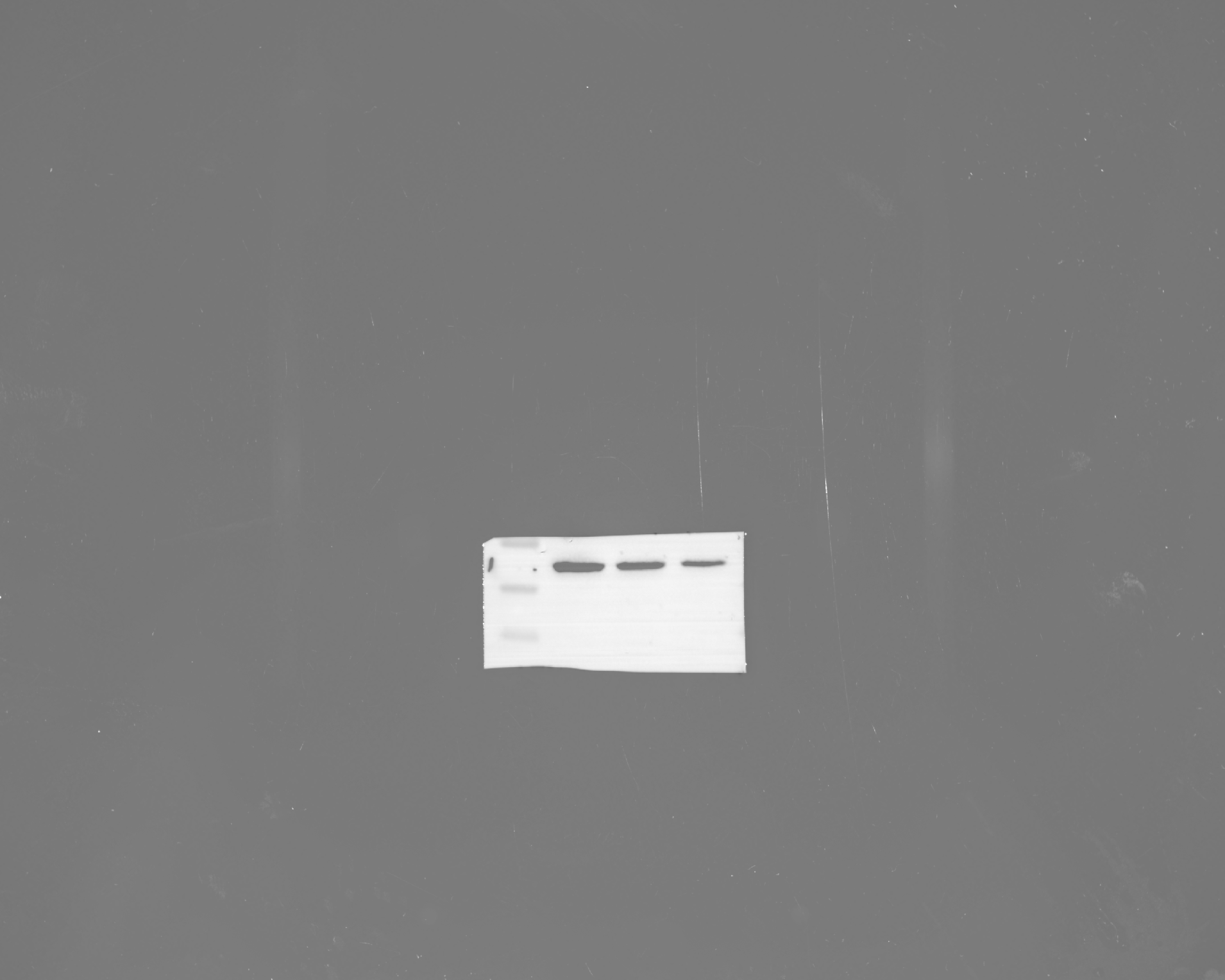

Supplement: Supplementary file 1 [file DataSheet1.zip › WB/HuH7 ZDHHC9.tif]

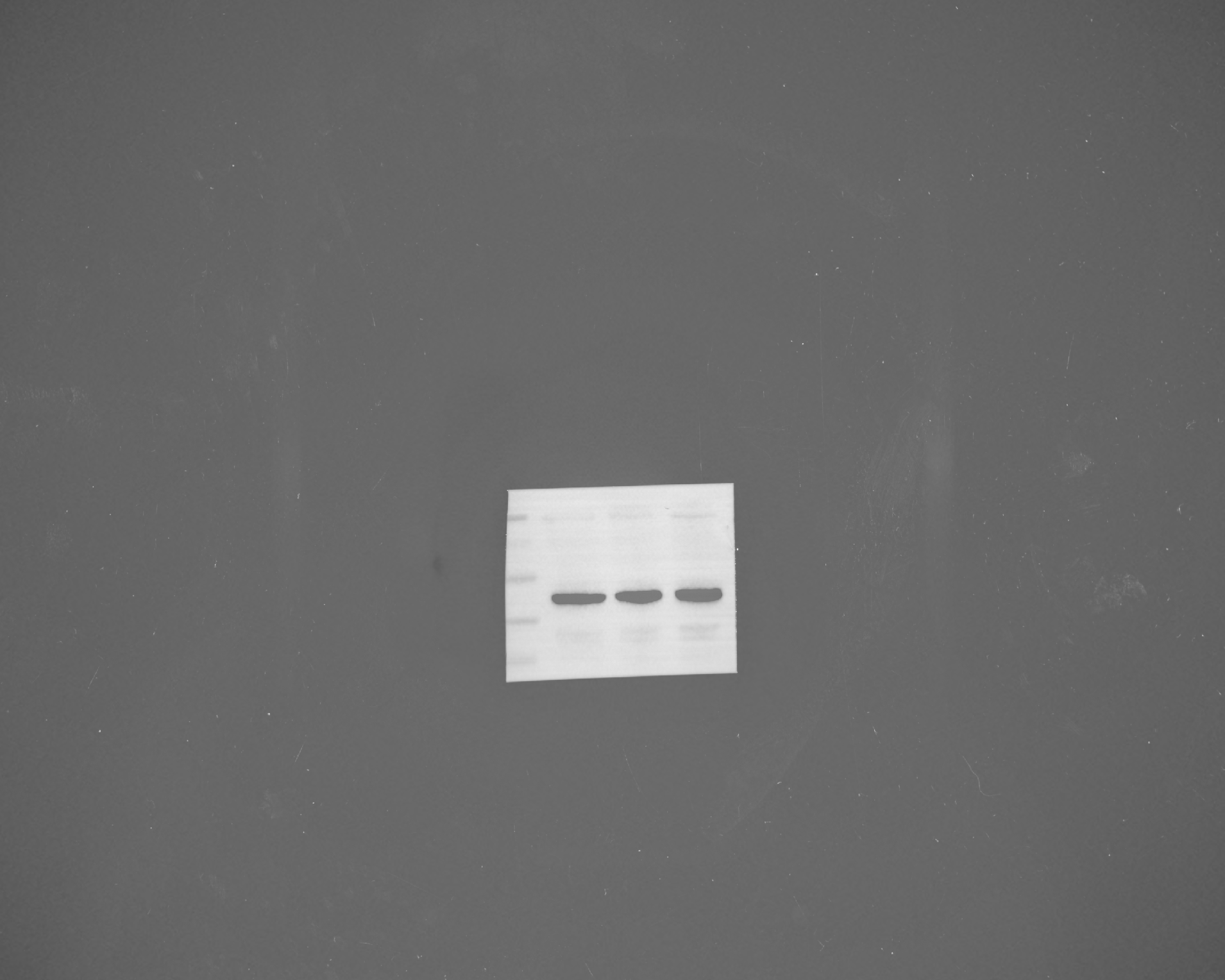

Supplement: Supplementary file 1 [file DataSheet1.zip › WB/HuH7 b-actin.tif]
