# Supplementary material for: Multi-omics and spatial transcriptomics decode the ZDHHC9-driven hypoxia-immunosuppressive axis in hepatocellular carcinoma
Source: Front Oncol. 2026 Jun 17;16:1869712. doi: 10.3389/fonc.2026.1869712 (PMC13318649; doi:10.3389/fonc.2026.1869712)
Supplement: Supplementary file 2 [file DataSheet2.docx]

**Multi-omics and Spatial Transcriptomics Decode the ZDHHC9-Driven Hypoxia-Immunosuppressive Axis in Hepatocellular Carcinoma**

**
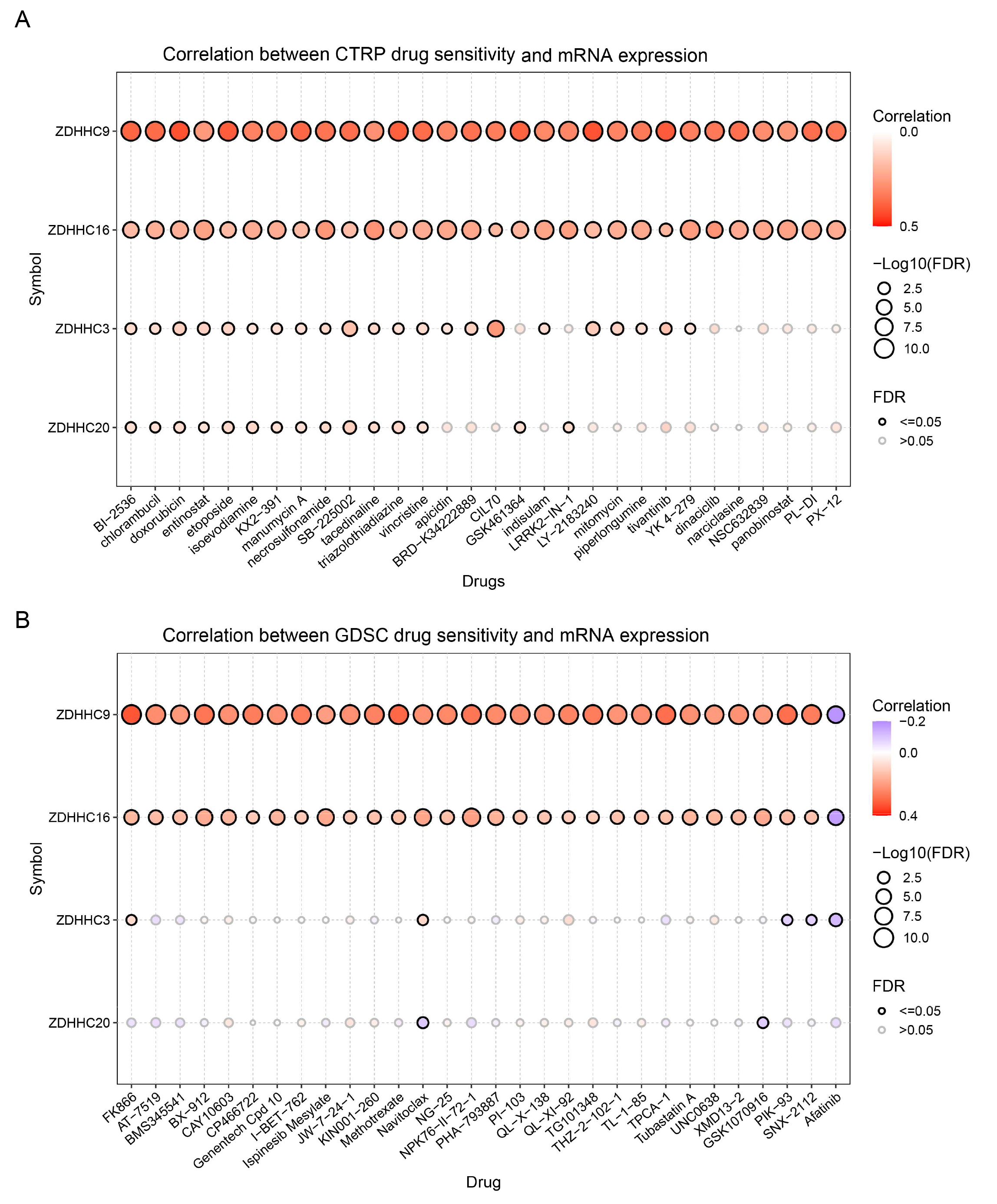
**

**Figure S1 Analysis of the correlation between ZDHHC3, ZDHHC9, ZDHHC16, and ZDHHC20 expression and drug sensitivity in various cancers.**

(A) The relationship between the expression of ZDHHC3, ZDHHC9, ZDHHC16, and ZDHHC20 and drug sensitivity across various cancers was examined using the GDSC database. (B) The association between the expression levels of ZDHHC3, ZDHHC9, ZDHHC16, and ZDHHC20 and drug sensitivity in different cancers was investigated through the CTRP database.

**
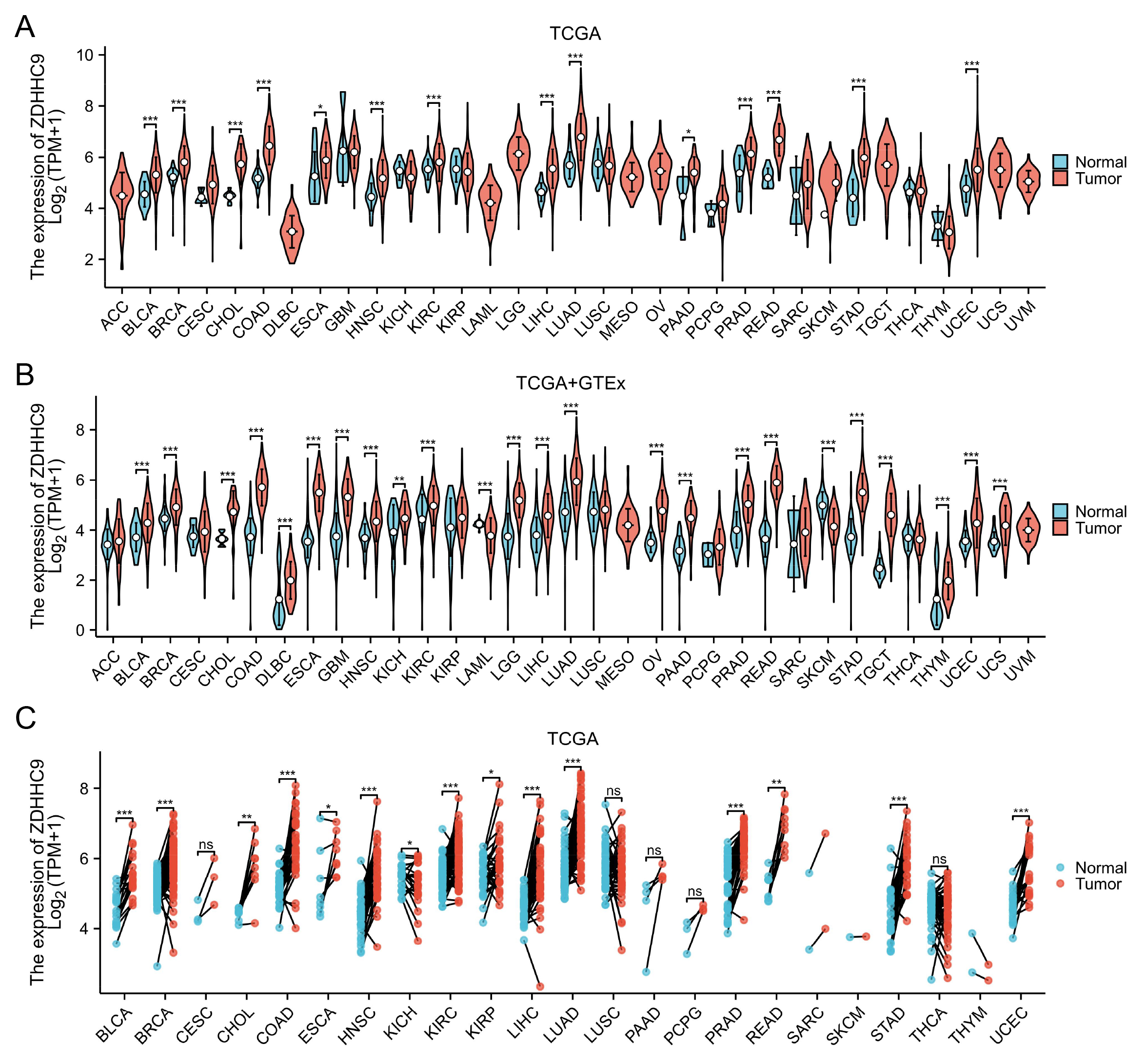
**

**Figure S2 Pan-cancer expression analysis of ZDHHC9.**

(A) Transcript levels of ZDHHC9 across tumor types in TCGA database. (B) Comparison of ZDHHC9 transcript levels between tumor tissues (TCGA) and normal tissues (GTEx). (C) Paired analysis of ZDHHC9 expression in tumor tissues versus matched adjacent normal tissues from TCGA. ns *P* ≥ 0.05, * *P* <0.05, ** *P* <0.01, *** *P* <0.001. Group comparisons in (A-C) were performed using the Wilcoxon rank-sum test.

**
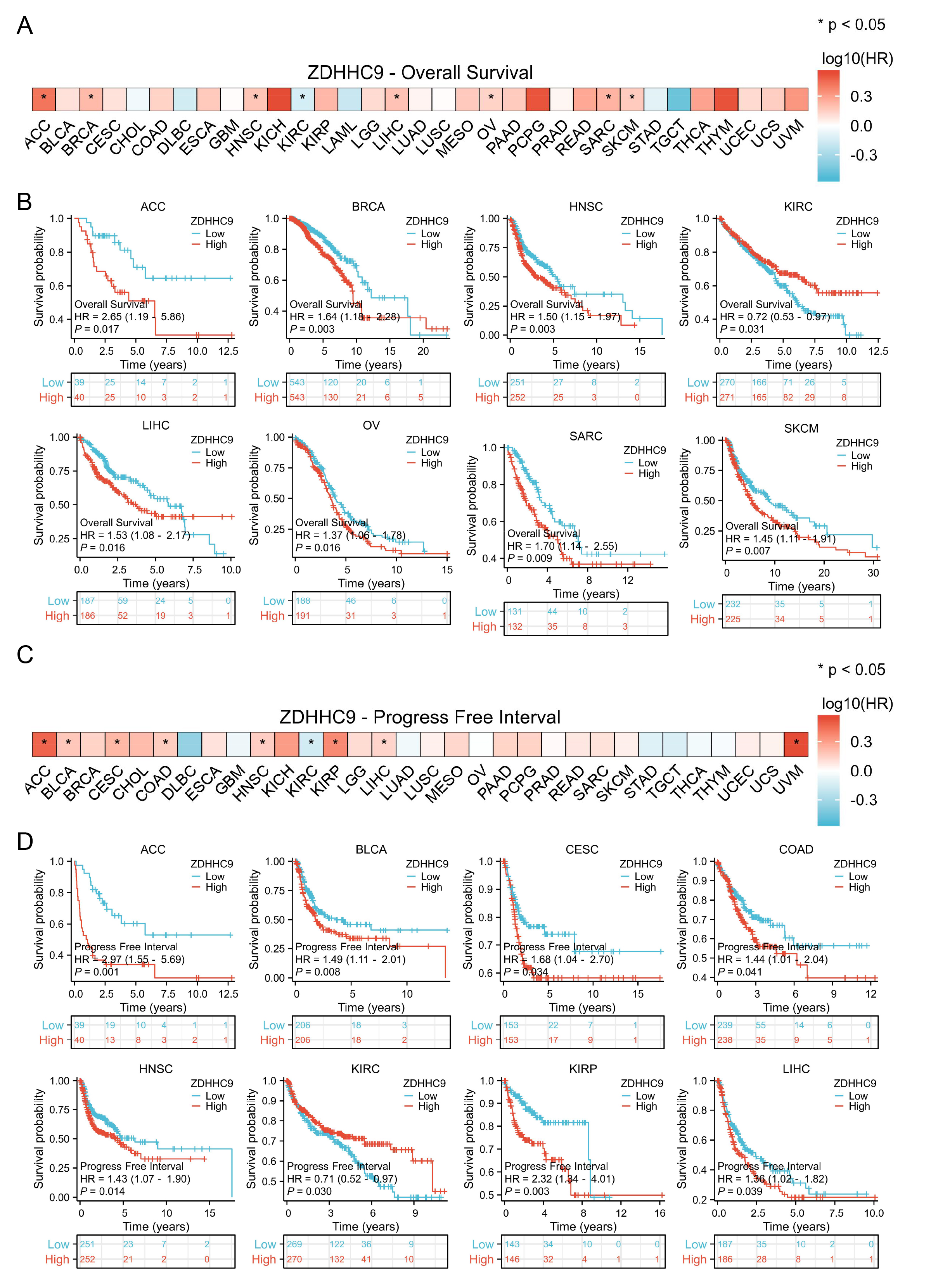
**

**Figure S3 Prognostic significance of ZDHHC9 across human cancers.**

(A) Heatmap depicting the association between ZDHHC9 expression and overall survival (OS) in pan-cancer analysis. (B) Representative Kaplan-Meier survival curves showing the correlation between ZDHHC9 expression levels and OS in selected cancer types. (C) Heatmap depicting the association between ZDHHC9 expression and progression-free interval (PFI) in pan-cancer analysis. (D) Representative Kaplan-Meier survival curves showing the correlation between ZDHHC9 expression levels and PFI in selected cancer types. * *P* <0.05, ** *P* <0.01, *** *P* <0.001. Statistical significance in (B, D) was determined by the log-rank test.


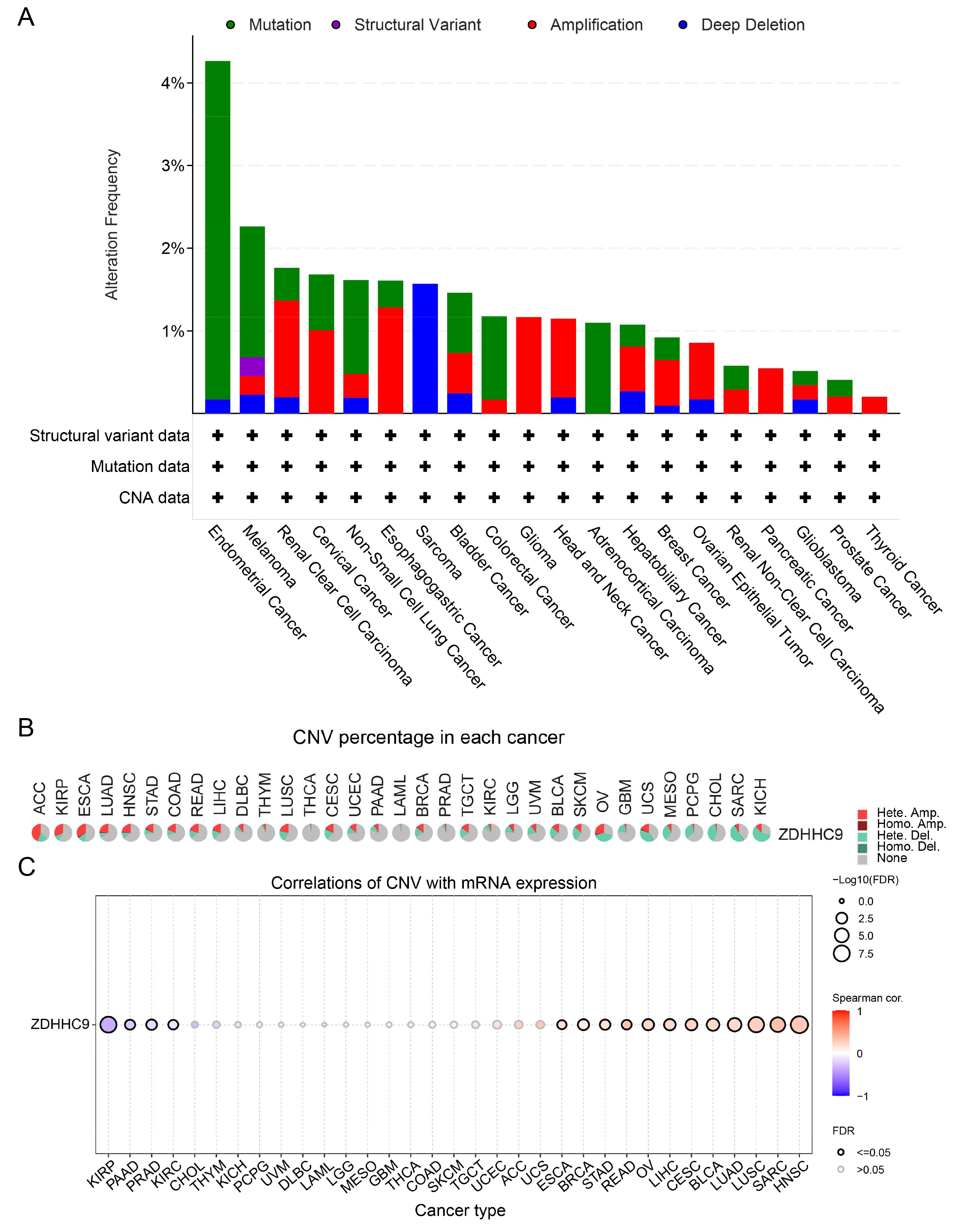


**Figure S4 Genomic alteration landscape of ZDHHC9 in human cancers.**

(A) Mutation frequency of ZDHHC9 across different cancer types (cBioPortal). (B) Impact of ZDHHC9 copy number variations (CNVs) on its mRNA expression levels in tumors. (C) Correlation analysis between ZDHHC9 expression levels and CNV status.


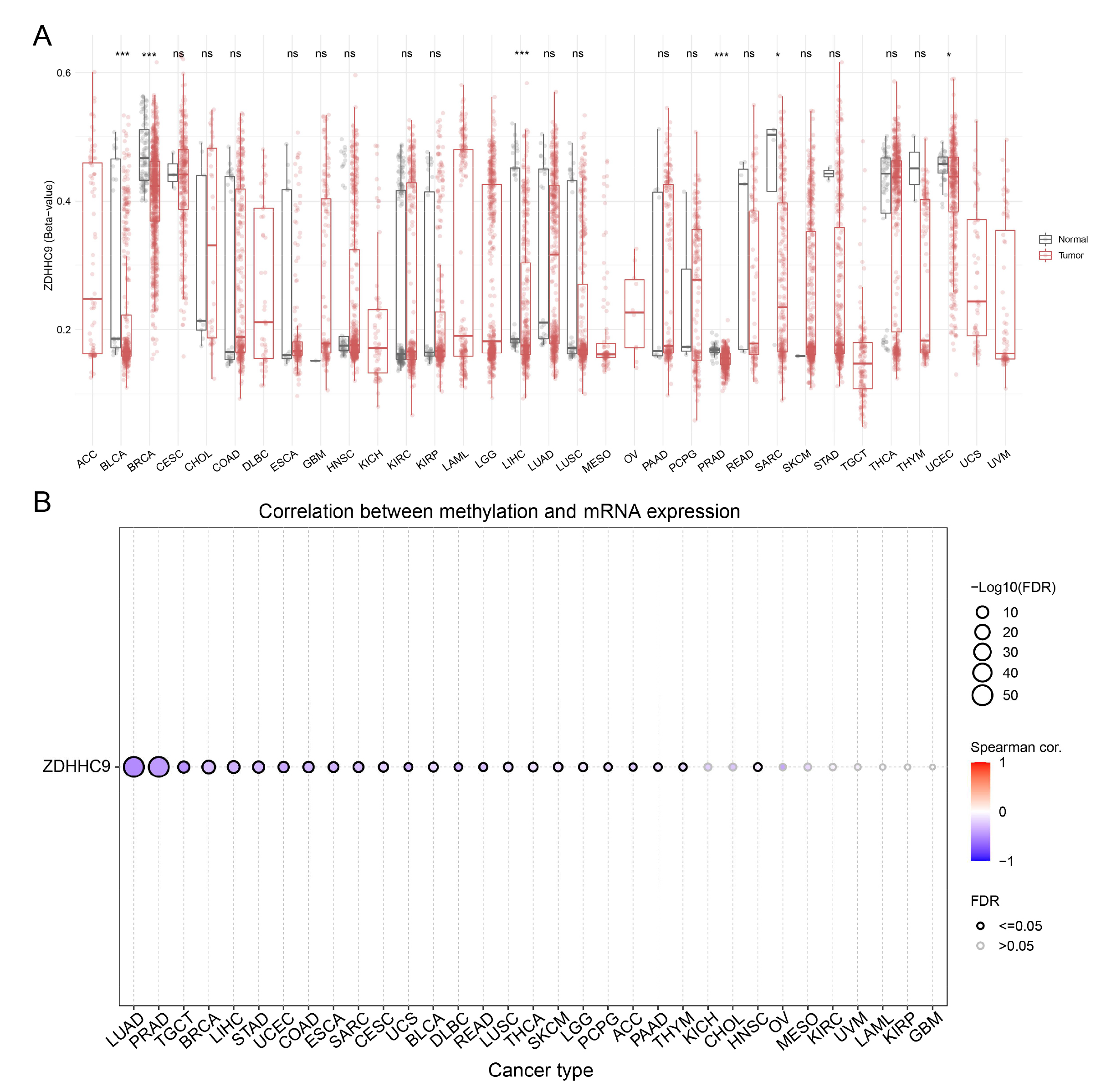


**Figure S5 DNA methylation regulation of ZDHHC9 in human cancers.**

(A) Differential DNA methylation levels at the ZDHHC9 gene promoter between tumor and normal tissues in multiple cancer types. (B) Correlation analysis between ZDHHC9 expression and its promoter DNA methylation levels. ns *P* ≥ 0.05, * *P* <0.05, ** *P* <0.01, *** *P* <0.001.

**
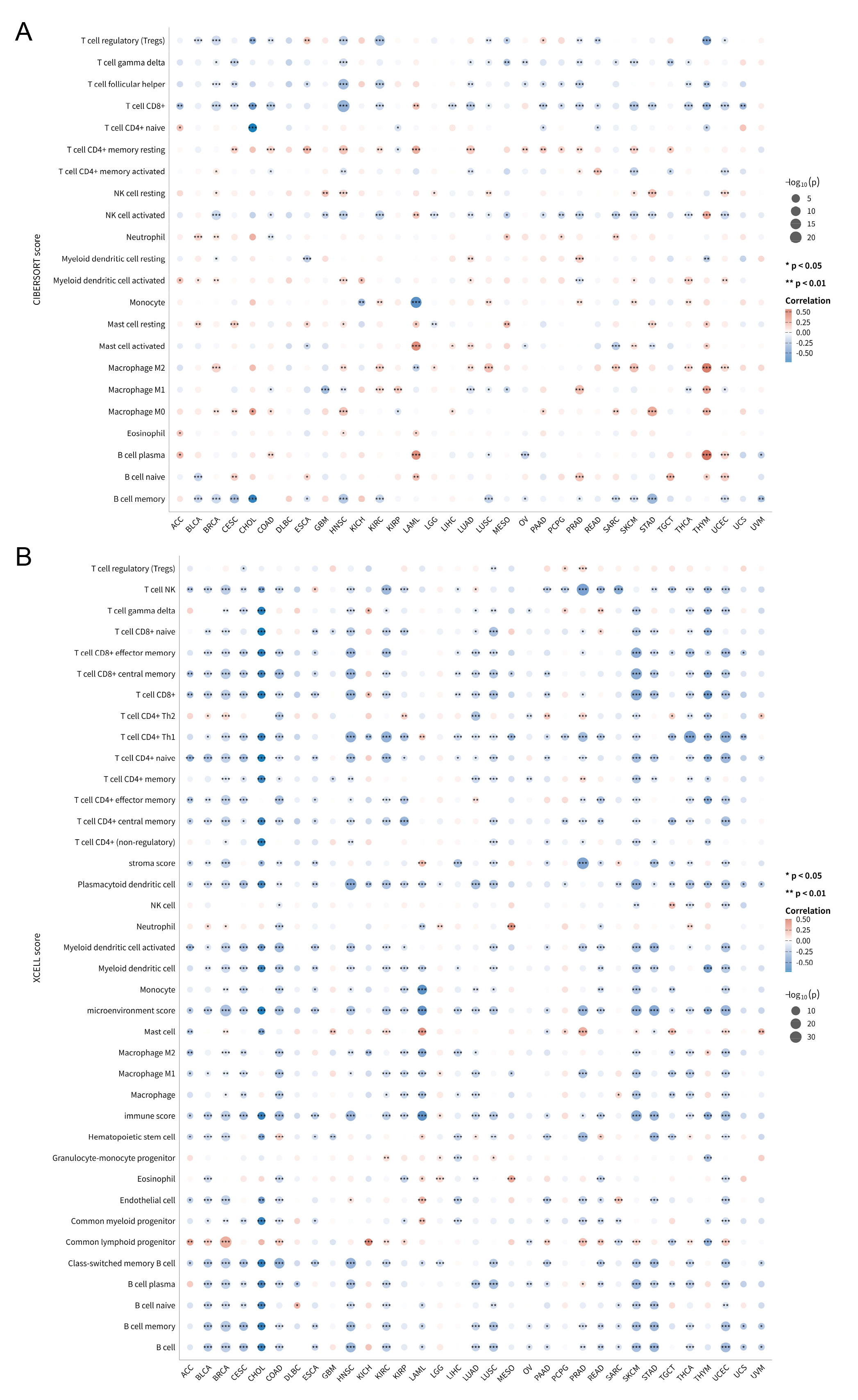
**

**Figure S6 Correlation between ZDHHC9 expression and tumor immune infiltration.**

(A-B) Heatmaps showing the correlation (Spearman's rho) between ZDHHC9 expression and the estimated abundance of six immune cell types across 30 cancer types (TIMER database). * *P* <0.05, ** *P* <0.01, *** *P* <0.001.


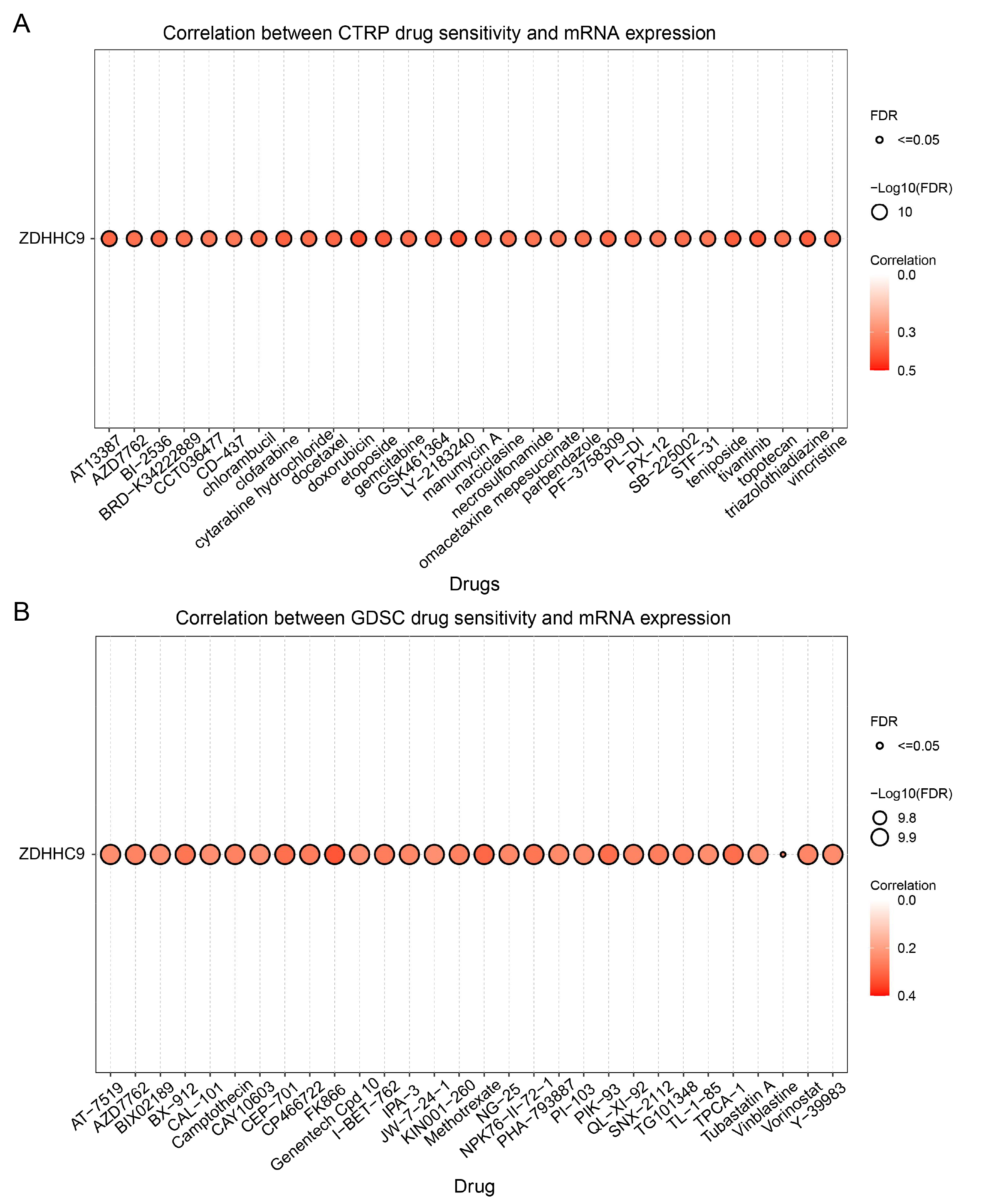


**Figure S7 Association of ZDHHC9 expression with drug sensitivity.**

(A-B) Drug sensitivity analysis of ZDHHC9 from CTRP and GDSC databases.
